# Emerging trends and hot spots of NLRP3 inflammasome in neurological diseases: A bibliometric analysis

**DOI:** 10.3389/fphar.2022.952211

**Published:** 2022-09-07

**Authors:** Xiaoyan Yu, Chuan Yu, Wenfang He

**Affiliations:** ^1^ Department of Critical Care Medicine, The Second Xiangya Hospital, Central South University, Changsha, China; ^2^ West China School of Public Health and West China Fourth Hospital, Sichuan University, Chengdu, China

**Keywords:** NLRP3 inflammasome, neurological diseases, inflammation, hot spots, trend, visualization analysis

## Abstract

**Background:** NLRP3 inflammasome has been of great interest in the field of neurological diseases. To visualize the research hotspots and evolutionary trends in this area, we collected the relevant articles in the Web of Science Core Collection database from 2010 to 2022 and analyzed them using CiteSpace software.

**Methods:** We performed a systematic search of the literature within the Web of Science Core Collection database using the strategy described below: TS = NLRP3 inflammasome AND TS = neurological diseases OR TS = neurological disorder OR TS = brain disorder OR TS = brain injury OR TS = central nervous system disease OR TS = CNS disease OR TS = central nervous system disorder OR TS = CNS disorder AND Language = English from 2010 to 2022. The type of literature was limited to articles and reviews. The data were processed using CiteSpace software (version 5.8. R3).

**Results:** A total of 1,217 literature from 67 countries/regions and 337 research institutions was retrieved. Publications in this area have increased rapidly since 2013. China presents the highest number of published articles, but the United States has a higher centrality and h-index. The top five most published institutions and authors are from China, Zhejiang University and Li Y ranking first, respectively. Of the ten most cited articles, Prof. Heneka MT and colleagues accounted for three of them. In terms of the co-occurrence keyword diagram, the five most frequent keywords are “nlrp3 inflammasome”, “activation”, “oxidative stress”, “expression”, and “alzheimers disease”.

**Conclusion:** The research of NLRP3 inflammasome in neurological disorders is overall developing well. Chinese scholars contributed the most significant number of articles, while researchers from developed countries presented more influential papers. The importance of NLRP3 inflammasome in neurological diseases is widely appreciated, and the mechanism is under study. Moreover, NLRP3 inflammasome is emerging as a promising therapeutic target in treating neurological disorders. However, despite decades of research, our understanding of NLRP3 inflammasome in central nervous system diseases is still lacking. More and more profound research is needed in the future.

## 1 Introduction

Inflammation is increasingly recognized as an important factor in neurological diseases. As an innate immune sensor, inflammasome has been of great interest in the field of immune and inflammatory diseases since its identification in 2002 by Jürg Tschopp’s research group (Martinon et al., 2002). The inflammasome is a protein complex containing the sensor protein (receptor), the adaptor apoptosis-associated speck-like protein containing a CARD (ASC), and the downstream effector caspase-1. Activation of receptor proteins by agonists subsequently recruits ASC and caspase-1 to assemble into inflammasomes up to micrometer in diameter, which induces caspase-1 self-cleavage and activation. Activated caspase-1 promotes the maturation and secretion of the pro-inflammatory cytokines interleukin-1β (IL-1β) and -18 (IL-18) on the one hand, and on the other hand, triggers cellular pyroptosis capable of clearing pathogens and damaged cells (Broz and Dixit, 2016). Depending on their receptors, the main inflammasomes include NLRC4 (NOD-like receptor family, CARD domain containing 4), NLRP1 (NOD-like receptor family, pyrin domain containing 1), NLRP3, AIM2 (absent in melanoma 2) and several others. NLRP3 inflammasome was first identified in 2004 by Jürg Tschopp and colleagues (Agostini et al., 2004) and is the best-described inflammasome in the central nervous system (CNS) at present. Its n-terminal protein-protein interaction structural domain is a PYRIN (PYD) and therefore requires the adaptor ASC (Agostini et al., 2004). Mutations of the *NLRP3* gene are responsible for the cryptochrome-associated periodic syndrome (CAPS) (Hoffman et al., 2001; Aganna et al., 2002; Aksentijevich et al., 2002).

NLRP3 inflammasome is associated with numerous CNS disorders, from sterile acute brain injuries to chronic neurodegenerative diseases (Walsh et al., 2014). The ability of the NLRP3 inflammasome to mediate various conditions and receive such high interest from researchers is closely related to its agonist properties. Unlike other members of inflammasomes which are activated only by one (or a few) specific agonists, NLRP3 inflammasome responds to many stimuli independent in origin, chemical composition, and structural properties. For CNS diseases, NLRP3 inflammasome is sensitive to endosome injury and aggregated proteins associated with diseases, including triphosphate (ATP) (Fann et al., 2018), amyloid-β (Aβ) (Halle et al., 2008), and α-synuclein (Panicker et al., 2019). It is suggested that the NLRP3 inflammasome may function as a signaling integrator, which recognizes any molecule or condition that would cause pathological states (Liston and Masters, 2017).

It is now well-recognized that the canonical activation of NLRP3 inflammasome is stimulated *via* two steps: prime (signal 1) and activation (signal 2). Briefly, signal 1 initiates the transcription of *NLRP3* and pro-inflammatory genes. Thus their expressions in cells are upregulated. This process implicates pattern recognition receptors (PRRs), such as tumor necrosis factor (TNF) receptors, nucleotide-binding oligomerization domain containing protein 2 (NOD2), and toll-like receptors (TLRs) triggered by various pathogen-associated molecular patterns (PAMPs) or damage-associated molecular patterns (DAMPs) (Franchi et al., 2009; Xing et al., 2017). Nuclear factor-κB (NF-κB) is then further activated and enhances the transcription of inflammasome-related components (Bauernfeind et al., 2009). Signal 2 occurs upon recognizing NLRP3 inflammasome stimuli and subsequently induces the assembly of ASC and caspase 1. However, it remains unclear how NLRP3 inflammasome sense and respond to cellular danger signals. The widely accepted upstream signals include K^+^ efflux *via* purinergic 2X7 receptor (P2X7R) (Munoz-planillo et al., 2013; Di et al., 2018), lysosomal disruption and the leakage of cathepsins (Hornung et al., 2008), production of reactive oxygen species (ROS) (Dostert et al., 2008), and mitochondrial dysfunction (Zhou et al., 2011; Iyer et al., 2013). ROS generation was consistently observed in the event of NLRP3 inflammasome activation, while inhibition of ROS production blocked the NLRP3 activation (Zhong et al., 2018). Damage to mitochondria leads to elevated ROS levels. It showed that impaired mitochondria could activate the NLRP3 inflammasome directly (Liu et al., 2018). Further study suggested that mitochondrial dysfunction was associated with high levels of ROS (He et al., 2016; Mills et al., 2018). Specific blockade of mitochondrial ROS production inhibited NLRP3 inflammasome activation (Zhou et al., 2011). Recently, it was revealed that the oxidized mitochondrial DNA (mtDNA) was required to activate the NLRP3 inflammasome, while loss of mtDNA in mice resulted in the inactivation of NLRP3 inflammasome (Zhong et al., 2018). Thus mitochondrial dysfunction and the liberation of mtROS and mtDNA into the cytoplasm is a key event associated with NLRP3 activation. Dysfunctions of other organelles such as the endoplasmic reticulum (Zhou et al., 2020) and Golgi apparatus (Chen and Chen, 2018) are also upstream events in the activation of NLRP3 inflammasome. Notably, these pathways are overlapping and cross-linked, and some studies yielded opposite conclusions. Further investigation is needed. In 2015, the remarkable identification of gasdermin D (GSDMD) linked to cellular pyroptosis was a breakthrough in the research field regarding NLRP3 inflammasome (Kayagaki et al., 2015). In general, when lipopolysaccharide (LPS) produced by Gram-negative bacteria is detected, caspases four and five in humans (Shi et al., 2014) [caspase 11 in mice (Kayagaki et al., 2011)] are activated *via* toll-like receptor 4 (TLR4), causing the cleavage of GSDMD, subsequently leading to K^+^ efflux and pyroptosis. This process is known as the non-canonical activation pathway of NLRP3 inflammasome. In the same year, another heavyweight article was published describing a selective NLRP3 inhibitor named MCC950 that further inspired a boom in inflammatory biotechnology (Coll et al., 2015). MCC950 does not inhibit any other recognized inflammasomes, including NLRP1, NLRC4, or AIM2. Meanwhile, MCC950 could inhibit both the canonical and non-canonical activation pathway induced by all currently known irritants of NLRP3 inflammasome (Coll et al., 2015). Because of its high specificity, MCC950 is the most recommended and used compound to investigate NLRP3 inflammasome-associated events *in vitro* and *in vivo*. Other small-molecule inhibitors of NLRP3 inflammasome include Bay 11–7082 (Juliana et al., 2010), JC-171 (Guo et al., 2017), etc.

With the increasing research on inhibitors of NLRP3 specificity, NLRP3 inflammasome is also thought to be a potential drug target for treating neurological diseases. In recent years, studies in this direction have grown rapidly. Bibliometric analysis can be applied to explore the developments of a specialty, mapping from its frontier to the knowledge base in a time-varying manner (Chen and Song, 2019). With the use of bibliometric software CiteSpace, our study intends to present realistic and intuitive pictures of the evolutionary trends of research hotspots in the field, and to assist researchers in better understanding of the research dynamics on NLRP3 inflammasome in neurological diseases.

## 2 Materials and methods

### 2.1 Data collection

**FIGURE 1:**
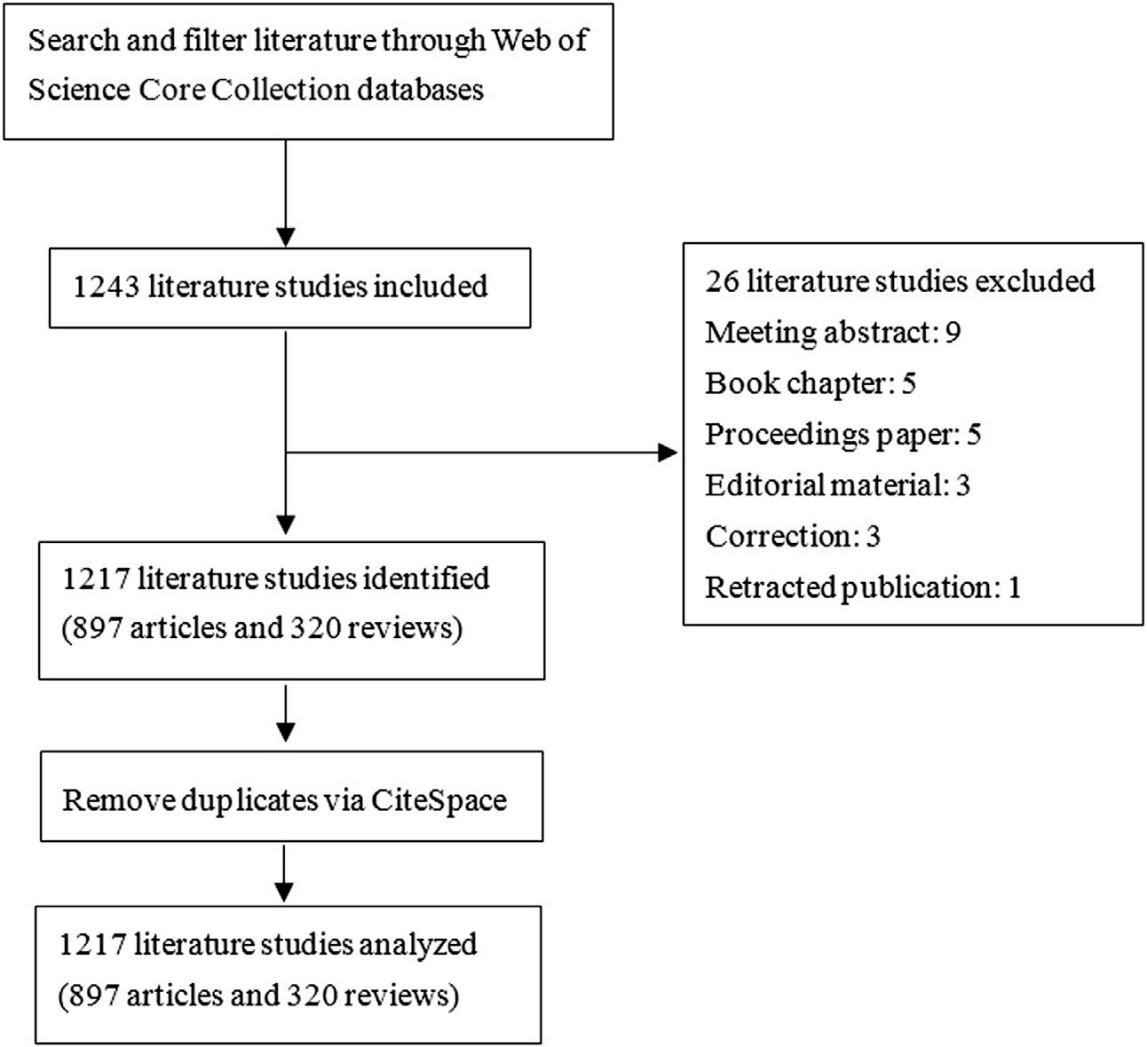
Schematic diagram of literature screening. Data collection was done on a single day, 12 May 2022. A total of 1,243 records from the Web of Science Core Collection (WoSCC) database was retrieved. Then, 26 data were excluded, including meeting abstracts, book chapters, proceedings papers, editorial material, corrections, and retracted publications. Finally, 1,217 pieces of data were obtained. Data were imported into the CiteSpace software (version 5.8. R3) for further analysis.

The initial systematic screening of the literature studies was performed within the Web of Science Core Collection (WoSCC) database. The searching strategy was as follows: TS = NLRP3 inflammasome AND TS = neurological diseases OR TS = neurological disorder OR TS = brain disorder OR TS = brain injury OR TS = central nervous system disease OR TS = CNS disease OR TS = central nervous system disorder OR TS = CNS disorder AND Language = English from 2010 to 2022. The type of literature was limited to articles and reviews. To avoid the impact of database updating, all data collection was done on a single day, 12 May 2022. We obtained a total of 1,243 records, and 26 data were excluded, including meeting abstracts, book chapters, proceedings paper, editorial material, corrections, and retracted publications. Data were imported into the CiteSpace software (version 5.8. R3) for further checking and processing. The recruitment strategy is shown in Figure 1.

### 2.2 Data analysis

The annual number of publications, publication years, h-index, and subject category were analyzed mainly through the WoSCC database. The h-index obtained from the WoSCC is a more accurate reflection of the academic achievements of an individual or a country/institution. A higher h-index indicates a higher impact of paper/scholar/country. The involved research institutions, countries and authors, keywords, and other indicators in this research field were analyzed by CiteSpace software. Graphs of contributed countries/regions, institutions, and authors were mapped. The co-occurring keywords, keyword cluster timeline, and keyword burst were also illustrated. Nodes in the generated map represent specific items, such as countries, institutions, keywords, and authors. The larger the node, the greater the number of documents. Links between nodes indicate cooperative networks. The thicker the line, the greater the intensity of cooperation. Centrality suggests the importance of the node in the networks. The centrality value >0.1 is generally regarded as a more significant node. The higher the co-occurrence frequency (Count) and centrality, the more influential the node is in the field. Keyword clustering timeline, co-occurrence keywords, and highly cited articles were used to identify the research status and focus. The keyword burst results reflect the dramatic increase in the hotness of a research direction over time and are applied to find current cutting-edge research topics.

## 3 Results

### 3.1 The global trend of publication outputs

**FIGURE 2:**
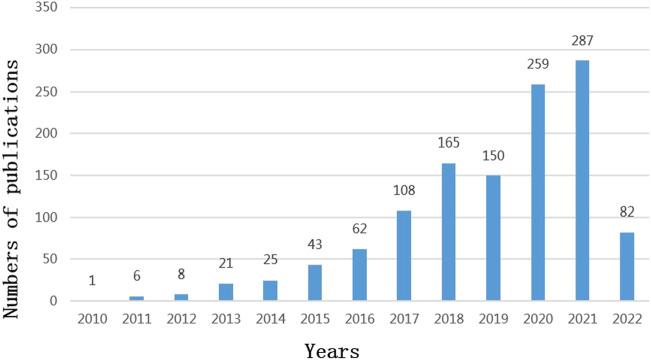
Publications over time (2010–2022).

A total of 1,217 articles were collected, including 897 articles and 320 reviews. 22302 articles were cited after removing self-citations. The total citation frequency excluding self-citation was 31577. The average citation frequency of each article was 31.04 times, and the h-index was 95. The annual number of publications is shown in Figure 2. Before 2013, the yearly literature in this field was in the single digits; it grew rapidly from 2013 onwards, with 287 publications by 2021. The main research areas include Neurosciences neurology (515, 42.32%), Immunology (258, 21.20%), and Pharmacology pharmacy (216, 17.75%), etc. The journals with the most published articles in this field are *Journal of Neuroinflammation* (70, 5.75%), *International Immunopharmacology* (38, 3.12%), *International Journal of Molecular Sciences* (37, 3.04%), *Frontiers in Immunology* (31, 2.55%), and *Brain Behavior and Immunity* (27, 2.22%). National Natural Science Foundation of China (NSFC) grants support the highest number of studies (416, 34.18%), followed by the National Institutes of Health (149, 12.24%) and the United States Department of Health Human Services (149, 12.24%) grants (Table 1).

**TABLE 1:** Top five based on the number of documents (2010–2022).

Field		Record count (%)
Research Areas	Neurosciences neurology	515 (42.32%)
	Immunology	258 (21.20%)
	Pharmacology pharmacy	216 (17.75%)
	Biochemistry molecular biology	197 (16.19%)
	Cell biology	145 (11.91%)
Journals	Journal of Neuroinflammation	70 (5.75%)
	International Immunopharmacology	38 (3.12%)
	International Journal of Molecular Sciences	37 (3.04%)
	Frontiers in Immunology	31 (2.55%)
	Brain Behavior and Immunity	27 (2.22%)
Funding Agencies	National Natural Science Foundation of China	416 (34.18%)
	National Institutes of Health (NIH United States)	149 (12.24%)
	United States Department of Health Human Services	149 (12.24%)
	NIH National Institute of Neurological Disorders Stroke	49 (4.03%)
	European Commission	48 (3.94%)

### 3.2 Contributions of countries and institutions

**FIGURE 3:**
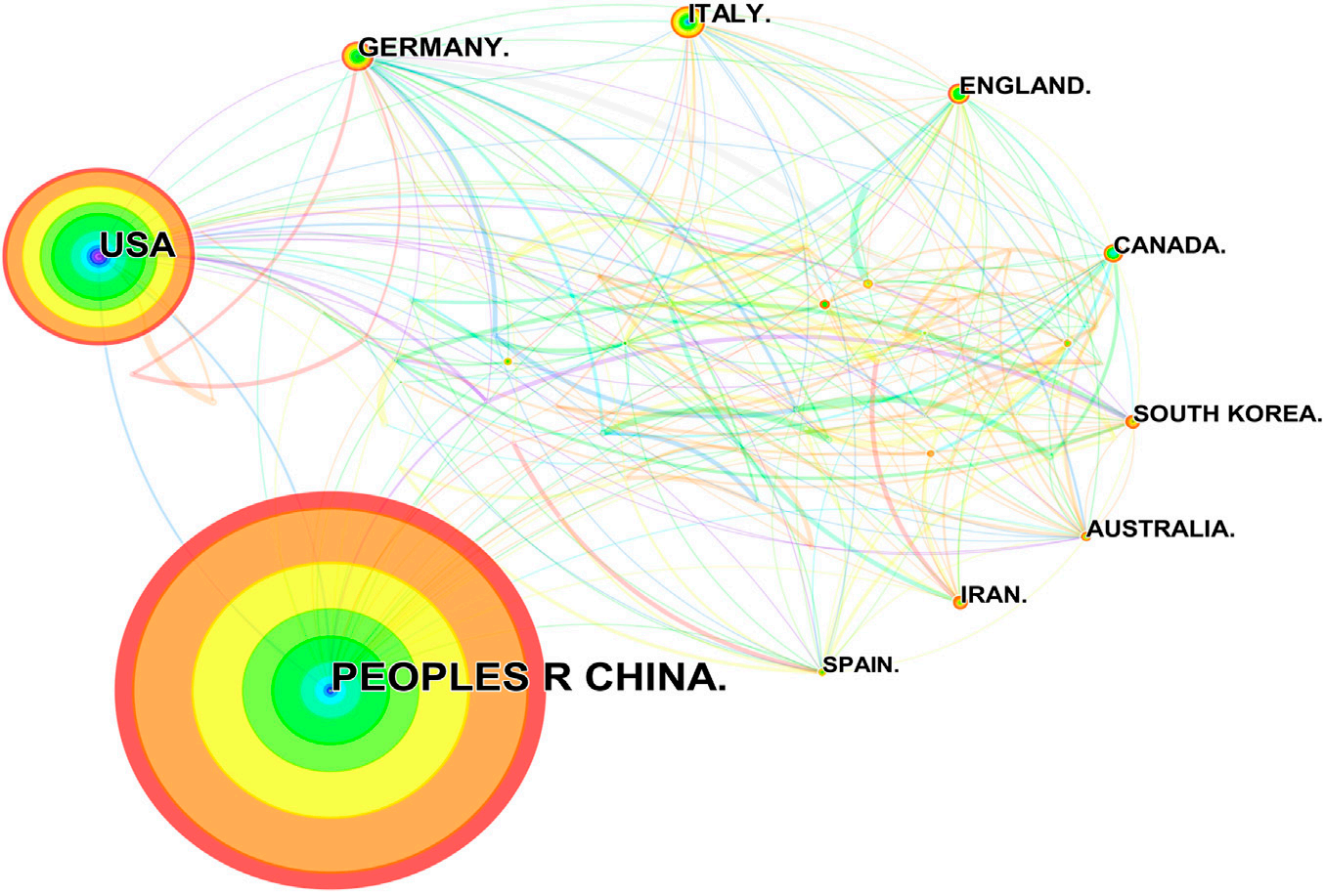
Publications over countries. The node size represents the number of articles. The larger the node, the greater the number of articles published by the country. The figure shows the top 20 countries in terms of number of published articles. China ranks first, and the other countries, in clockwise order, are the United States, Germany, Italy, England, Canada, South Korea, Australia, Iran, and Spain. The lines between the nodes represent the cooperation between the different countries.

**FIGURE 4:**
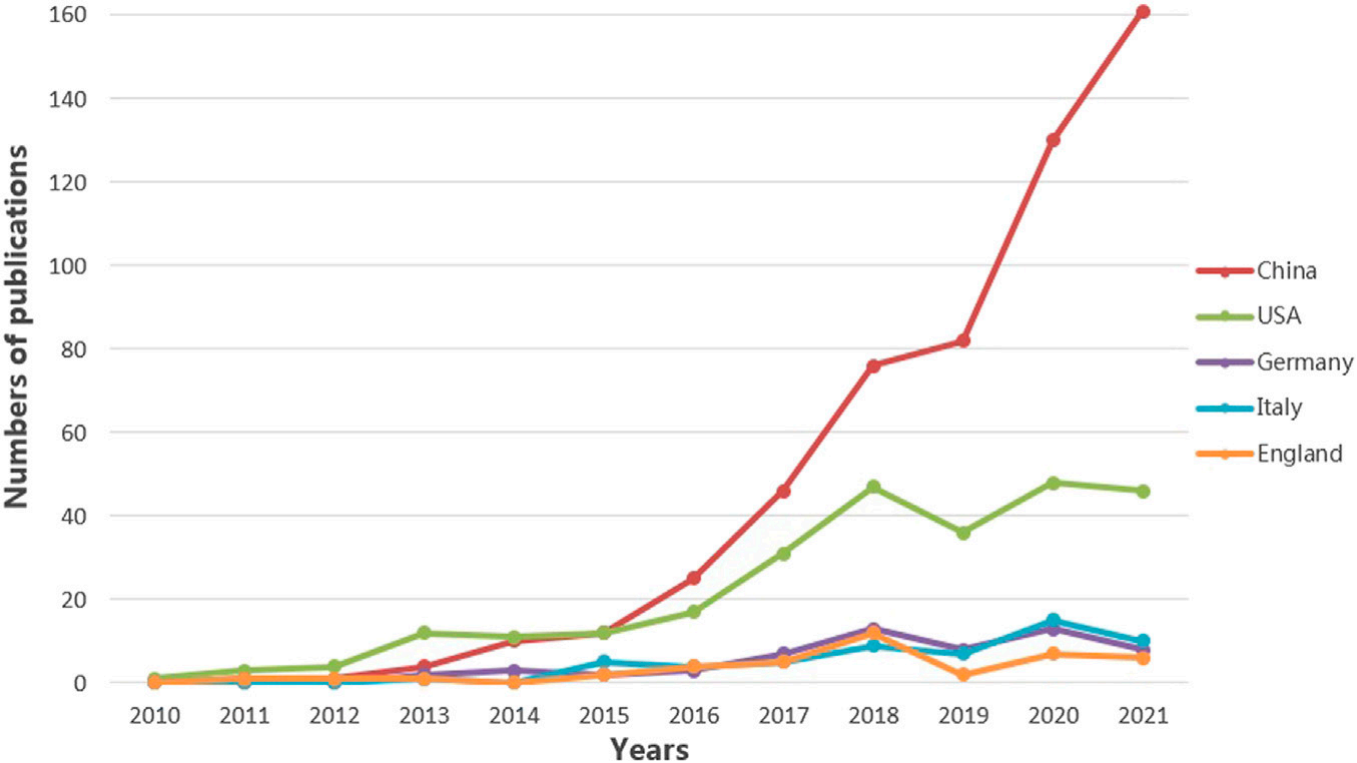
Annual number of publications over countries. The five countries with the highest number of published articles are China, the United States, Germany, Italy, and England. The annual number of publications from these five countries is illustrated. The different color lines represent different countries.

**FIGURE 5:**
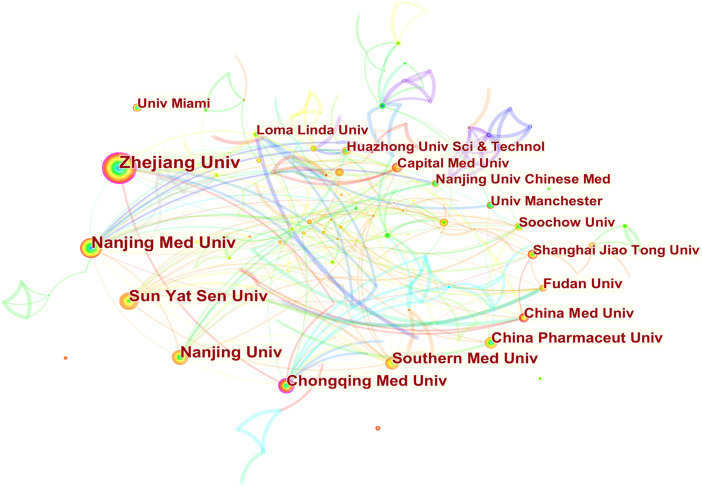
Publications over institutions. The node size represents the number of articles. The larger the node, the greater the number of articles. The highest number of publications is from Zhejiang University, followed by Nanjing Medical University, Sun Yat Sen University, Nanjing University, and Chongqing Medicine University. Other countries are shown in counterclockwise order in the picture. The lines between the nodes represent the cooperation between the different institutions.

Researchers from 67 countries/regions and 337 institutions were involved in the studies in this field. As shown in Table 2, among the 67 countries/regions, those contributing more literature are China (604, 49.63%), the United States of America (USA) (280, 23.01%), Germany (64, 5.26%), Italy (59, 4.85%), and England (45, 3.70%). Figure 3 visualizes the outputs and connections between different countries. Links among nodes reveal that there was active cooperation among countries. The size of the node indicates the number of publications. The purple outer circle of the node shows that the centrality of the node is more significant than 0.1, which means that this node is critical in the network. Of note, although China ranks first in the number of published articles, the centrality is less than the United States, suggesting that the latter is more important in collaborative networks. Germany has fewer articles but higher centrality, indicating more collaboration. The annual publications for each country are shown in Figure 4. As shown in Table 2 and Figure 5, the top five institutions in terms of the number of articles are all from China, namely Zhejiang University (44, 3.62%), Nanjing Medical University (33, 2.71%), Sun Yat Sen University (28, 2.30%), Nanjing University (25, 2.05%) and Chongqing Medicine University (24, 1.97%). However, the generated map showed 337 nodes and only 445 links (Figure 5), indicating less cooperation between institutions.

**TABLE 2:** Top five countries/regions and institutions based on the number of documents (2010–2022).

Field		Record count (%)	Centrality	h-index
Countries	China	604 (49.63%)	0.17	57
	United States	280 (23.01%)	0.36	66
	Germany	64 (5.26%)	0.21	34
	Italy	59 (4.85%)	0.16	24
	England	45 (3.70%)	0.12	22
Affiliations	Zhejiang University	44 (3.62%)	0.12	21
	Nanjing Medical University	33 (2.71%)	0.10	20
	Sun Yat Sen University	28 (2.30%)	0.05	12
	Nanjing University	25 (2.05%)	0.07	16
	Chongqing Medicine University	24 (1.97%)	0.10	13

### 3.3 Authors and co-cited authors

A total of 438 authors have participated in the research on the role of NLRP3 inflammasome in neurological diseases. As shown in Table 3, the five most prolific authors are, in rank order, Li Y (Shanghai Jiao Tong University, count 16), Wang J (Soochow University, count 15), Wang Y (Zhejiang University, count 14), Chen S (Zhejiang University, count 14), and Wang L (Zhejiang University, count 14). Surprisingly, all five authors are from China but have relatively low centrality. More interestingly, most of them conducted their research post 2016.

**TABLE 3:** Top five authors and co-cited authors (2010–2022).

Authors			
Name	Affiliations	Count	First appearance year
Li Y (Li Yi)	Shanghai Jiao Tong University	16	2018
Wang J (Wang Jian)	Soochow University	15	2016
Wang Y (Wang Yan)	Zhejiang University	14	2016
Chen S (Chen Sheng)	Zhejiang University	14	2013
Wang L (Wang Lin)	Zhejiang University	14	2016
Co-cited authors			
Name	Affiliations	Citation	Centrality
Heneka MT	University of Bonn (Germany)	277	0.03
Martinon F	Université Paris-Saclay (France)	235	0.02
Schroder K	The University of Queensland (Australia)	205	0.00
Zhou Rongbin	University of Science and Technology of China (China)	195	0.03
Halle A	Center of Advanced European Studies and Research (Germany)	181	0.06

**FIGURE 6:**
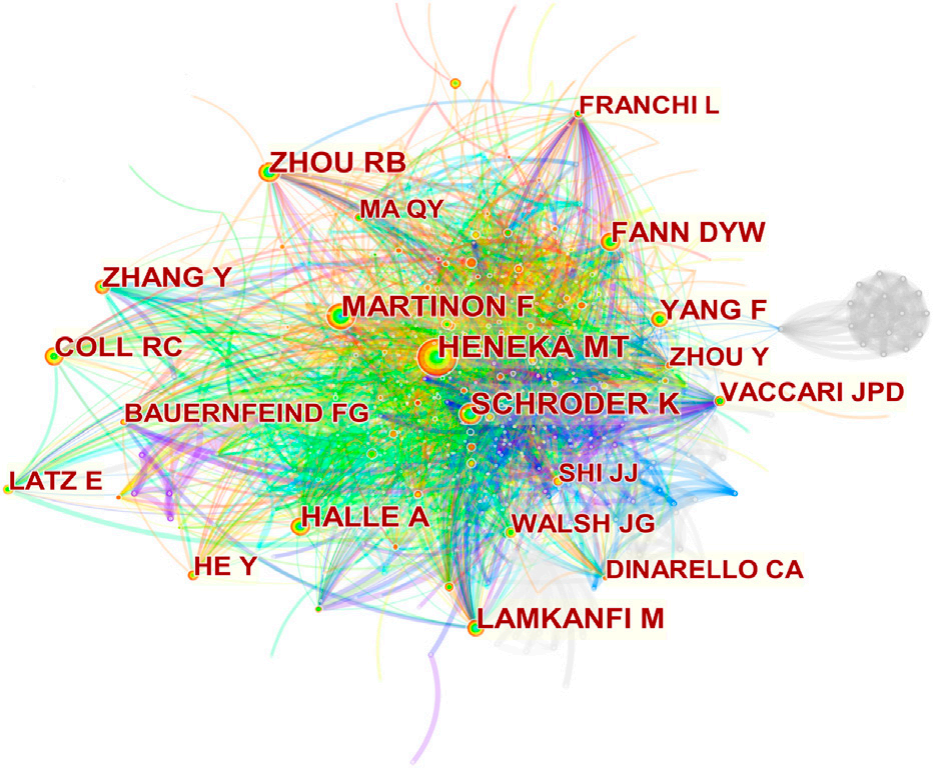
Co-cited authors’ network. Nodes represent co-cited authors. The size of the node represents the number of citations. The line between nodes represents the collaboration between authors.

A single co-citation is considered when two or more authors are cited at the same time in a single paper. These two or more authors are co-cited authors and form a co-citation network. Of 746 co-cited authors, 18 are co-cited more than 100 times. Heneka MT (277) is the most frequently co-cited author, followed by Martinon F (235) (Table 3). Figure 6 presents the top 20 co-cited authors and shows relatively close cooperation between authors.

### 3.4 Research topic and frontiers

#### 3.4.1 Top 10 highly cited references

Highly cited literature refers to publications of high citation frequency and impact, reflecting the hot spots and depth of research in the area. Table 4 lists the top ten most cited articles on NLRP3 inflammasome in CNS disease research from 2010 to 2022. Interestingly, half of these ten articles were released in 2013. Among these ten papers, the article published in *Nature* by Heneka MT and colleagues ranks top, with a remarkable 1,385 citations times. This article, along with the second-, fifth-, sixth-, and seventh-ranked literature, discussed the specific mechanisms of NLRP3 inflammasome signaling axis in the pathogenesis of Alzheimer’s disease (AD) and other neurodegenerative diseases. The ninth- and tenth-ranked articles addressed the involvement of NLRP3 inflammasome in stroke, suggesting the potential clinical value of therapeutic interventions targeting inflammasome assemblies and activities. The third and fourth-ranked articles summarized the understanding of NLRP3 inflammasome in the genesis of neurological disorders. These findings demonstrate the intense interest of researchers over the past decade regarding the role of NLRP3 inflammasome in the pathogenesis of neurological diseases and potential therapeutic targets.

**TABLE 4:** Top 10 high-cited references related to NLRP3 inflammasome in neurological diseases.

Ranking	Title	Authors	Journal	Year	Citation
1	NLRP3 is activated in Alzheimer’s disease and contributes to pathology in APP/PS1 mice	Heneka MT; Kummer MP; Golenbock DT et al.	NATURE	2013	1,385
2	Innate immune activation in neurodegenerative disease	Heneka MT; Kummer MP and Latz E et al.	NATURE REVIEWS IMMUNOLOGY	2014	833
3	Inflammasomes in the CNS	Walsh JG; Muruve DA and Power C	NATURE REVIEWS NEUROSCIENCE	2014	399
4	Cytokines in Inflammatory Disease	Kany S; Vollrath JT and Relja B	INTERNATIONAL JOURNAL OF MOLECULAR SCIENCES	2019	379
5	Microglia-derived ASC specks cross-seed amyloid-beta in Alzheimer’s disease	Venegas C; Kumar S; Heneka MT et al.	NATURE	2017	367
6	Canonical Nlrp3 Inflammasome Links Systemic Low-Grade Inflammation to Functional Decline in Aging	Youm YH; Grant RW; Dixit VD et al.	CELL METABOLISM	2013	344
7	Triggering of Inflammasome by Aggregated alpha-Synuclein, an Inflammatory Response in Synucleinopathies	Codolo G; Plotegher N; de Bernard M et al.	PLOS ONE	2013	318
8	The inflammasome: Pathways linking psychological stress, depression, and systemic illnesses	Iwata M; Ota KT and Duman RS	BRAIN BEHAVIOR AND IMMUNITY	2013	314
9	Intravenous immunoglobulin suppresses NLRP1 and NLRP3 inflammasome-mediated neuronal death in ischemic stroke	Fann DYW; Lee SY; Arumugam TV et al.	CELL DEATH & DISEASE	2013	277
10	Functions and mechanisms of microglia/macrophages in neuroinflammation and neurogenesis after stroke	Xiong XY; Liu L and Yang QW.	PROGRESS IN NEUROBIOLOGY	2016	272

#### 3.4.2 Keyword co-occurrence

**FIGURE 7:**
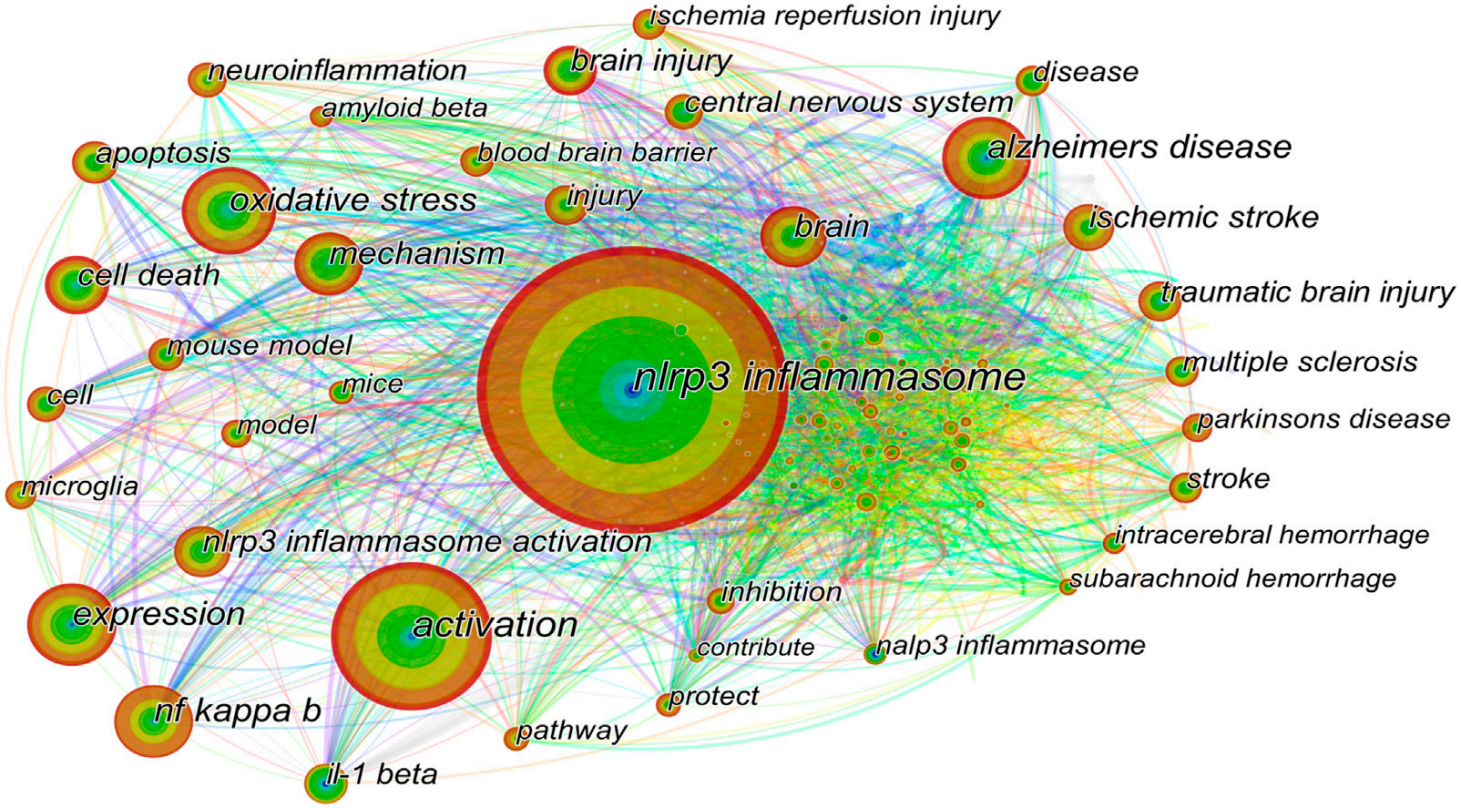
Keywords co-occurrence network. The size of the node represents the count of keywords. The top ten co-occurrence keywords are “nlrp3 inflammasome”, “activation”, “oxidative stress”, “expression”, “alzheimers disease”, “nf kappa b”, “mechanism”, “brain”, and “cell death”. The right part of the figure illustrates the major diseases associated with NLRP3 inflammasome, including “alzheimers disease”, “ischemic stroke”, “traumatic brain injury”, and “multiple sclerosis”. The keywords related to the NLRP3 inflammasome activation pathways are shown in the lower-left corner of the figure, such as “nf kappa b” and “il-1 beta”. Keywords related to the mechanism of NLRP3 inflammasome activation are indicated in the upper-left corner of the figure, including “oxidative stress”, “apoptosis”, and “neuroinflammation”.

The keyword co-occurrence map is based on the frequency of keyword co-occurrence in the cited literature, namely, two or more keywords appearing in the same literature are considered as one co-occurrence. Keyword co-occurrence analysis assists in identifying research hotspots and predicting research trends in certain areas. The keywords with high co-occurrence frequency have been illustrated in Table 5. The top ten co-occurrence keywords are “nlrp3 inflammasome” (595), “activation” (306), “oxidative stress” (190), “expression” (181), “alzheimers disease” (181), “nf kappa b” (171), “mechanism” (149), “brain” (140), and “cell death” (134). To facilitate understanding, we have drawn a keyword co-occurrence network map (Figure 7). The right panel of the chart illustrates the major diseases associated with NLRP3 inflammasome, in addition to “alzheimers disease”, including “ischemic stroke”, “traumatic brain injury”, “multiple sclerosis”, “parkinsons disease”, “intracerebral hemorrhage” and “subarachnoid hemorrhage”. The keywords associated with NLRP3 inflammasome activation pathways are represented in the lower-left corner of the map, such as “nf kappa b” and “il-1 beta”. Keywords related to the mechanism of NLRP3 inflammasome are indicated in the upper-left corner of the figure, including “oxidative stress”, “apoptosis”, “neuroinflammation”, and “blood brain barrier”.

**TABLE 5:** Keywords co-occurrence frequency (Top 25 in count order, 2010–2022).

Keywords	Count	Centrality
nlrp3 inflammasome	595	0.06
activation	306	0.08
oxidative stress	190	0.05
expression	181	0.03
alzheimers disease	181	0.06
nf kappa b	171	0.06
mechanism	149	0.05
brain	140	0.04
cell death	134	0.02
nlrp3 inflammasome activation	121	0.03
ischemic stroke	121	0.05
brain injury	121	0.04
il-1 beta	107	0.07
injury	103	0.03
apoptosis	102	0.01
central nervous system	102	0.08
traumatic brain injury	92	0.03
neuroinflammation	92	0.02
cell	85	0.02
disease	79	0.01
multiple sclerosis	77	0.03
mouse model	77	0.03
stroke	75	0.02
inhibition	74	0.01
blood brain barrier	73	0.03

#### 3.4.3 Keyword cluster timeline and keyword burst

**FIGURE 8:**
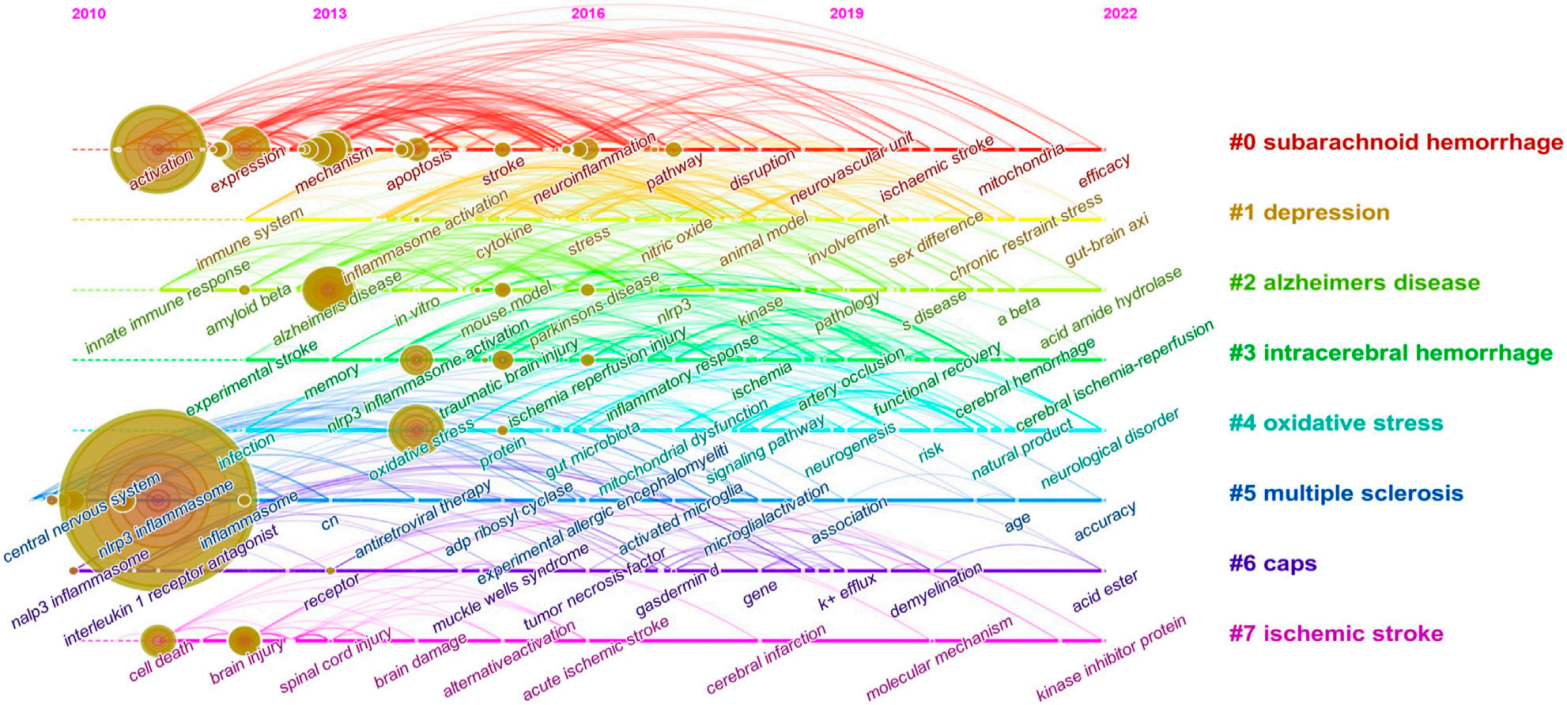
Keyword cluster timeline analysis (2010–2022). There are eight clusters in total and distinguished by different colors. Cluster #0 is the largest cluster, followed by cluster #1, and so on. This timeline chart spreads out the keywords included in the clusters by the time they emerged. The keyword color is the same as the label color of the cluster to which it belongs.

**FIGURE 9:**
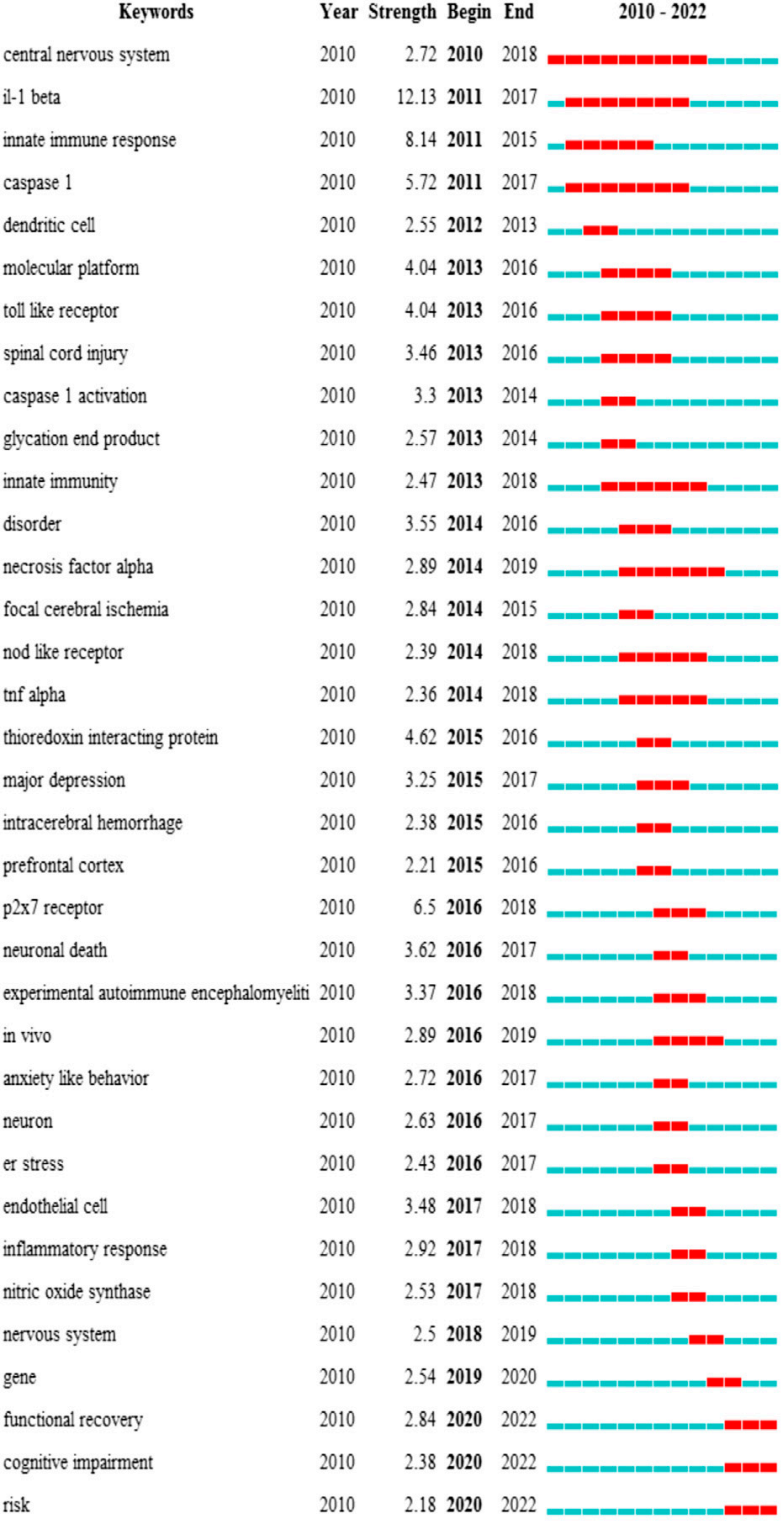
Top 35 keywords with the strongest citation burst. The burst results suggest a spike in citations for a specific keyword during a certain period. In other words, relevant research has attracted a high degree of attention in the field. It is an indicator of the research frontier within a specific time. The blue line indicates the period, and the red line indicates the duration of the citation burst. The number in parentheses represents the intensity of the burst. The larger the number, the higher the intensity of the burst.

Keyword cluster refers to the network group based on keyword co-occurrence with similar research topics. A total of eight clusters are generated: “subarachnoid hemorrhage”, “depression”, “alzheimers disease”, “intracerebral hemorrhage”, “oxidative stress”, “multiple sclerosis”, “caps”, and “ischemic stroke”. In addition, in CiteSpace, clusters are numbered starting from zero. That is, cluster #0 is the largest cluster, while cluster #1 is the second-largest, and onwards. The keyword timeline after clustering is drawn using TimeLine View (Figure 8). In the TimeLine View, these keywords are spread out in their respective clusters according to the years they emerged. The keyword color is consistent with the label color of the cluster to which it belongs. The length of the horizontal solid line for each cluster represents its timeframe. The TimeLine View visually reveals the historical span of the literature and is used to follow the evolution of research trends. We further drew the graph of keyword citation burst using CiteSpace (Figure 8), with the blue line indicating the period and the red line indicating the duration of the citation burst, showing the progress of the hot topics. As shown in Figure 9, “il-1 beta” (12.13) has the highest strength, followed by “innate immune response” (8.14) and “caspase 1” (5.72). Keywords with a long duration of citation burst include “central nervous system” (2010–2017), “il-1 beta” (2011–2017), “caspase 1” (2011–2017), “innate immune response” (2011–2015), “innate immunity” (2013–2018), and “necrosis factor alpha” (2014–2019), indicating that studies in these directions received a lot of attention from researchers. The latest burst keywords include “functional recovery” (2020–2022) and “cognitive impairment” (2020–2022).

## 4 Discussion

### 4.1 Research trends

As shown in Figure 2, it is clear that the number of studies in this field has shown a continuous increase since 2013. It is also apparent from Table 4 that there were many heavyweight papers released in 2013, as represented by the article published in *Nature* by Prof. Heneka MT’s team (Heneka et al., 2013). In addition, most of the highly cited papers were published in top journals, such as *Nature* and *Cell,* demonstrating the significant interest and recognition of researchers. These impressive findings have certainly stimulated the interest of other scholars in the NLRP3 inflammasome. Thus not surprisingly, the number of articles has grown since then. As presented in Table 2, China, the United States, Germany, Italy, and England are the top five contributing countries in terms of the number of publications. Of note, China has contributed almost half of the papers published in this field. The top five institutions with the highest number of releases, all from Chinese universities, are Zhejiang University, Nanjing Medical University, Sun Yat Sen University, Nanjing University, and Chongqing Medicine University. The top five contributing authors are also from China: Li Y, Wang J, Wang Y, Chen S, and Wang L. Among these five authors, the last three authors are based at Zhejiang University. Interestingly, three of these five authors launched their studies in 2016. The fund that supports the most research is NSFC. Thus, our data illustrate the consistency among productive authors, leading countries and institutions, and investing funds. Because of the ample financial support, the intense academic atmosphere in excellent institutions, and the high productivity of authors, China surpassed the United States in terms of publications in 2016 and has continued its leadership position (Figure 4).

It is worth noting that although the United States ranks second in overall publications, it outperforms China to rank first in terms of h-index. Meanwhile, we also recognize that Chinese authors accounted for only one of the highly cited articles and ranked ninth, which is relatively backward. There are many reasons for the insufficient impact of Chinese scholars’ articles, probably related to the later start of Chinese academics’ studies. Chinese researchers should keep deepening their investigation in the field to enhance their influence later on. In addition, there is less collaboration between China and other countries (Figure 3). It is expected that cooperation between countries and institutions can be strengthened in the future to promote progress together. In contrast, Germany publishes fewer articles, but its collaboration with other countries remains intense. Among the top ten highly cited literature, Prof. Heneka MT and colleagues contributed three papers. As the Head of the Department of Neurodegenerative Diseases and Geriatric Psychiatry at the University Hospital Bonn, and the Head of the Research Group of the German Center for Neurodegenerative Diseases (DZNE), from 2013 till today, he has continued to focus and conduct research on NLRP3 inflammasome in AD, with several high-quality publications and a significant influence in the field. Therefore it is reasonable for him to be a highly co-cited author.

### 4.2 Research focuses and frontiers

Summarized from the keywords burst chart (Figure 9), research on NLRP3 inflammasome in CNS diseases can be roughly divided into three stages. The first phase from 2010 focused on the expression and activation of the NLRP3 inflammasome signaling pathway in CNS disease. Therefore, downstream potency factors in the signaling pathway, such as “caspase1” and “il-1 β”, were hot spots for keywords. This was followed by a second phase from 2013 onwards, in which the specific mechanisms of NLRP3 inflammasome involved in certain CNS diseases were the focus of researchers’ attention. Thus, both related mechanisms and diseases showed explosive growth in this period, including “disorder”, “focal cerebral ischemia”, “intracerebral hemorrhage, and “er stress”. Immediately following the third phase in 2020, more attention was paid to the feasibility of NLRP3 inflammasome as a therapeutic target, so keywords regarding the evaluation of the efficacy to treat diseases became popular, such as “functional recovery” and “cognitive impairment”. Of note, as shown in TimeLine View (Figure 8), these three stages are not developed separately but are intertwined and share mutual progress. Currently, NLRP3 inflammasome activation is under investigation in a wide range of neurological disorders. In particular, disorders including neurodegenerative diseases, hemorrhagic/ischemic stroke, and related mechanisms such as oxidative stress, receive long-standing concerns. Similar conclusions can be drawn from the keyword co-occurrence graph (Figure 7). As presented in Table 1, the research directions of NLRP3 inflammasome include not only Neurosciences neurology but also Immunology and Pharmacology, which also reflect the focus and interest of researchers.

As discussed in the previous section, the canonical activation of NLRP3 requires two steps: priming (signal 1) and activation (signal 2). At present, the primary mechanism of signal 1 is relatively well established. However, the mechanism of signal 2 is still not elucidated. Therefore, as shown in Figure 7, related studies are the hot spot in this field. Besides K^+^ efflux, Ca^2+^ release from the endoplasmic reticulum (ER) promoted by K^+^ is also thought to be an upstream signal in NLRP3 inflammasome activation (Murakami et al., 2012). More specifically, ER stress leading to Ca^2+^ release from the ER lumen amplifies the activation of NLRP3 inflammasome. This may partly contribute to why ER stress has recently become popular (Figure 9). In addition, mitochondrial impairment, and the release of mtROS and mtDNA into the cytoplasm are critical upstream events associated with NLRP3 inflammasome activation (Shimada et al., 2012). MtROS levels are significantly elevated in response to cellular stress. Considering the essential role of oxidative stress in the activation of NLRP3 inflammasome and in mediating the pathogenesis process of neurological diseases such as stroke, not surprisingly, it has become a hot topic of research.

Ischemic stroke is the second leading cause of death worldwide (Campbell et al., 2019). Cognitive impairment and depression are major complications of strokes, resulting in heavy social and medical burdens (Pendlebury and Rothwell, 2009). The ninth and tenth most highly cited literature, and several other studies, highlighted the importance of NLRP3 inflammasome in the inflammatory response in aseptic tissue involved in acute brain injury after stroke (Fann et al., 2013a; Fann et al., 2013b; Xiong et al., 2016). NLRP3 inflammasome and pro-inflammatory cytokines such as IL-1 β and caspase-1 were highly expressed in cellular and animal models of stroke, as well as in stroke patients (Fann et al., 2013a; Barrington et al., 2017). The production of mtROS induced by ischemia/reperfusion (I/R) could activate the NLRP3 inflammasome and lead to neural damage (Minutoli et al., 2016). Other danger signals in I/R that activate NLRP3 inflammasome include mitochondrial DNA oxidation, mitochondrial dysfunction, intracellular Ca^2+^ overloading, cell swelling, and thioredoxin-interacting protein (TXNIP) (Gong et al., 2018; Feng et al., 2020). TXNIP is dissociated from the thioredoxin 1 (Trx1)/TXNIP complex in oxidative stress conditions. It was further suggested that during rats’ I/R injury, the Trx1/TXNIP complex was modulated by nuclear factor erythroid 2-related factor 2 (Nrf2), an important transcription factor involved in the antioxidant stress system (Hou et al., 2018). Nrf2 scavenges ROS and then inhibits NLRP3 expression (Liu et al., 2017). After upregulation of Nrf2, the expression of cytoplasmic TXNIP, NLRP3 inflammasome, and downstream elements caspase-1, IL-18, and IL-1β were remarkably decreased while knockdown of Nrf2 resulted in the opposite (Hou et al., 2018). Therefore, Nrf2 could function as a protective mediator of NLRP3 inflammasome activation, which may represent an innovative therapeutic insight for ischemia treatment. Currently, it is well recognized that the inflammatory cascade mediated by NLRP3 inflammasome can lead to brain edema, hemorrhage, blood-brain barrier injury, and more neuronal death (Wang et al., 2007). The possible mechanisms involved include promoting the maturation and release of pro-inflammatory factors and mediating apoptosis (Fann et al., 2013b). In line with this, further studies have shown that the blockade of NLRP3 inflammasome activation serves a neuroprotective function (Fann et al., 2013a) (Yang et al., 2014). Treatments with P2X7R antagonist blue brilliant G (BBG) or MCC950 to inhibit the activation of NLRP3 inflammasome decreased infarct volume and neurovascular complications in the mouse model of middle cerebral artery occlusion (MCAO) and suppressed neuronal cell apoptosis *in vitro* and *in vivo* (Ye et al., 2017). Furthermore, treatment of MCC950 could alleviate cognitive impairment, blood-brain barrier integrity, and neurovascular remodeling in rats after ischemia (Ward et al., 2019; Bellut et al., 2021).

NLRP3 inflammasome can also be activated after hemorrhagic stroke, such as intracerebral hemorrhage (ICH) and subarachnoid hemorrhage (SAH), leading to an inflammatory response cascade that further exacerbates brain injury (Luo et al., 2019b). A previous study showed that P2X7R/cryopyrin inflammatory axis contributed to the activation of caspase-1 and the subsequent production of IL-1 β/IL-18 after SAH, thus promoting neuroinflammation (Chen et al., 2013). In a mouse model of SAH caused by endovascular perforation, the pro-inflammatory mediator triggering receptor expressed on myeloid cells 1 (TREM-1) was identified to induce microglia NLRP3 inflammasome activation and the following pyroptosis (Xu et al., 2021). In contrast, TREM-1 inhibition ameliorated neurological deficits. Intraperitoneal injection of MCC950 in rats attenuated neuroinflammatory response, reduced early brain injury, and improved SAH-induced neurological dysfunction (Dodd et al., 2021; Luo et al., 2019a). MCC950 treatment in ICH murine models could also preserve the integrity of the blood-brain barrier, reduce the generation of pro-inflammatory cytokines by microglia, and decrease neuronal cell death (Ren et al., 2018). BBG treatment attenuated neuroinflammation after ICH in rats by inhibiting the P2X7R/NLRP3 inflammasome pathway (Feng et al., 2015). Nrf2 also plays a protective role by attenuating early brain injury in ICH (Luo et al., 2019b). Silymarin upregulated the Nrf2-mediated signaling pathway that led to the inactivation of NLRP3 inflammasome, exerting neuroprotective effects in rats with collagenase-induced ICH (Yuan et al., 2017).

Multiple sclerosis (MS) is a progressive inflammatory demyelinating and incurable disease that affects more than 2.3 million people worldwide, causing motor, sensory, vision, cognitive, autonomic, and mood disorders (Thompson et al., 2018). To date, the cause of MS is unknown. Experimental autoimmune encephalomyelitis (EAE), featured by CD4^+^ T cell-mediated inflammation and demyelination, is an ideal model for human MS. Several studies demonstrated that NLRP3 inflammasomes were required in the development of EAE (Inoue et al., 2012a; Lalor et al., 2011). Loss of NLRP3 inflammasome and the downstream effector IL-1β and IL-18 ameliorated the course of EAE by reducing T-cell initiation and later T-cell trafficking to the central nervous system (Inoue et al., 2012a; Lalor et al., 2011). Jenny P.-Y. Ting and colleagues demonstrated that, for the first time, mice lacking the *Nlrp3* gene (*Nlrp3* −/−) exhibited reduced demyelination in EAE models (Jha et al., 2010). Similar results were also observed in *caspase 1*−/− and *IL-18*−/− mice, suggesting that NLRP3 inflammasome was involved in the pathological process of MS *via* caspase 1 and IL-18 (Jha et al., 2010). Notably, *IL-18−/−* mice presented enhanced myelin regeneration. Thus intervention to inhibit IL-18 may be a future target for demyelinating diseases. With these articles, a landmark explosion of research regarding NLRP3 inflammasome on MS occurred afterward (Figure 8). Interventional inhibition of NLRP3 inflammasome did show therapeutic effects on MS in animal models. As a specific inhibitor of the NLRP3 inflammasome, MCC950 was first used in the study of EAE after its development. MCC950 treatments reduced IL-1β production, postponed the onset, and mitigated the severity of EAE in mice (Coll et al., 2015). These results were verified by Malhotra S and colleagues recently. EAE models of mice treated with MCC950 exhibited reduced disease severity and decreased LPS-induced axonal damage (Malhotra et al., 2020). In both *in vitro* and *in vivo* EAE models, JC-171, another small-molecule inhibitor of the NLRP3 inflammasome, proved to reduce NLRP3-mediated IL-1β secretion in a dose-dependent manner by disrupting NLRP3-ASC interactions (Guo et al., 2017). A recent study showed that treatment with another small-molecule inhibitor, VX-765, could reduce the expression of IL-1β, caspase 1, and GSDMD in EAE models, and yielded superior outcomes, including prevention of axonal injury and improved neurobehavioral function (Mckenzie et al., 2018). This finding further suggested that GSDMD-mediated pyroptosis was involved in the mechanism of the inflammatory demyelination process in MS, which was previously unsuspected. Manoalide is a newly discovered selective NLRP3 inhibitor that acts mainly by blocking the NEK7-NLRP3 interaction. Manoalide intervention mitigated the progression of EAE and disease severity by alleviating the neuroinflammatory response in EAE mice (Li et al., 2022). Moreover, recent studies also suggested that *NLRP3* gene polymorphism could predict the interferon beta (IFN-β) efficacy in MS patients’ treatment (Malhotra et al., 2015; Imani et al., 2018; Inoue et al., 2012b). After IFN-β administration, increased expression of NLRP3 and IL-1β was observed in responders while no changes in non-responders (Malhotra et al., 2015). However, this proposition is still controversial. In contrast, a meta-analysis conducted by Sunny Malhotra and colleagues suggested that NLRP3 polymorphisms were not associated with the response to IFN-β treatment in MS (Malhotra et al., 2018). Research on this subject is still ongoing, which may partially explain why the keyword “gene” has been prevalent in recent years.

AD is a specific neurodegenerative disease that affects up to 6.5 million Americans (Alzheimer’s Disease International, 2022). About two decades ago, Halle A and colleagues demonstrated that, for the first time, Aβ could directly activate the NLRP3 inflammasome and lead to the release of IL-1β (Halle et al., 2008). The probable mechanisms included lysosomal damage and the following cathepsin B release (Halle et al., 2008). Prof. Halle A also ranked the fifth most highly co-cited author (Table 3). Subsequently, extensive research was conducted. The outstanding representative of this area is the article published by Prof. Heneka MT and colleagues, which tops the citation list (Table 4). It demonstrated that Nlrp3 (−/−) or Casp1 (−/−) mice were largely free of spatial memory loss and other sequelae related to AD, and exhibited decreased cerebral caspase-1 and IL-1β activation, along with increased Aβ clearing (Heneka et al., 2013). In the amyloid precursor protein/presenilin 1 (APP/PS1) AD model, NLRP3 inflammasome defects would shift microglia toward the M2 phenotype, leading to reduced Aβ deposition (Heneka et al., 2013). This research team further revealed that NLRP3 inflammasome activation was related to ASC-speck cross-seeding of Aβ pathology in AD mice models, suggesting the essential role of NLRP3 inflammasome in the genesis and development of Aβ pathology (Venegas et al., 2017). The latest study showed that in addition to fibrillar Aβ aggregates, NLRP3 inflammasome could be activated by lower molecular weight Aβ oligomers and protofibrils (Luciunaite et al., 2020). This finding highlighted the probability that microglia activation by these Aβ species might trigger the innate immune response ahead of the onset of Aβ deposition. Recently, Prof. Heneka MT and colleagues further confirmed that activation of microglia NLPR3 inflammasome could promote tau hyperphosphorylation and aggregation and facilitate the onset of AD (Ising et al., 2019). Meanwhile, NLRP3 inflammasome can be directly activated by microglia tau oligomers, monomers (Ising et al., 2019), and aggregates (Stancu et al., 2019), mediating Aβ-induced tau cascade pathology (Ising et al., 2019). Moreover, NLRP3-deficient mice exhibited reduced tau pathology, and improved cognitive function (Ising et al., 2019). These discoveries suggest the significant role of NLRP3 inflammasome in the pathogenesis of AD and, more importantly, indicate that patients with AD may benefit from therapeutic strategies targeting NLRP3 inflammasome. Extensive studies have shown that MCC950 treatment could decrease Aβ-induced pathological events and improve cognitive functions. MCC950 could suppress the NLRP3 inflammasome-mediated immune response induced by Aβ aggregates (Luciunaite et al., 2020) and the tau-induced pathological changes (Stancu et al., 2019). The inhibition of NLRP3 inflammasome by MCC950 improved synaptic plasticity deficits *in vivo* (Qi et al., 2018), and ameliorated memory impairment in murine models (Fekete et al., 2019). Other inhibitors of NLRP3 inflammasome also exhibited neuroprotective effects, with the ability to improve neuronal damage and cognitive impairment, including Bay 11–7082 (Ruan et al., 2019) and VX-765 (Flores et al., 2018).

As shown in Figure 7 and Table 5, traumatic brain injury (TBI) has also gained popularity among researchers in recent years. TBI induces an inflammatory cascade that includes potassium efflux, altered calcium signaling, mitochondrial damage, and ROS release, all of which ultimately trigger NLRP3 inflammasome activation (O’brien et al., 2020). In the acute phase of the TBI animal model, MCC950 intervention preserved the integrity of the blood-brain barrier, attenuated NLRP3-mediated neuroinflammatory response, and improved disease outcome (Ismael et al., 2018; Xu et al., 2018). Bay 11–7082 also significantly reduced inflammatory infiltration and damage in mouse cortex and hippocampus within 24 h after TBI (Irrera et al., 2017). The study further showed that NLRP3-deficient mice exhibited retained cognitive function and less severe brain damage when compared to wild-type mice.

### 4.3 Research in the future

With so much evidence from animal studies, NLRP3 inflammasome is emerging as a new target for treating CNS diseases. However, no drugs directly targeting NLRP3 inflammasome are available for clinical use in neurological disorders at present. Anakinra is one of three IL-1 inhibiting biologics approved by the U.S. Food and Drug Administration (FDA) for therapeutic use in multiple inflammatory diseases. As a reconstituted IL-1 receptor antagonist, anakinra was the first to be developed and effective in the treatment of CAPS (Jesus and Goldbach-mansky, 2014) and rheumatoid arthritis (Calabrese, 2002). Although anakinra therapy showed benefits in preclinical models of neurological disorders, several issues limit its practical clinical application. Anakinas requires subcutaneous injection daily due to their short plasma half-life (Granowitz et al., 1992). However, it causes inflammation at the injection site, making daily administration very painful (Rossi-semerano et al., 2015). More importantly, blocking IL-1β signaling of all sources would increase the risk of infection, which has long been noted in anakinra’s application for rheumatoid arthritis (Galloway et al., 2011; Ramirez and Canete, 2018). Subsequently, the emergence of selective small-molecule inhibitors of NLRP3 inflammasome offers new possibilities. Regrettably, a phase II clinical trial of MCC950 for rheumatoid arthritis was halted due to hepatotoxicity (Mackenzie et al., 2010). Meanwhile, the clinical trial of VX-765 was also suspended owing to its immunosuppression and hepatic toxicity (Mackenzie et al., 2010). Therefore, there is still considerable interest in identifying a small molecule NLRP3 inhibitor that has the potential of increasing efficacy, reducing side effects, and could be used to manage NLRP3-related neuroinflammatory and CNS disorders. Other NLRP3 inflammasome-specific inhibitors, including inzomelid (Inflazome/Roche) and NT-0167 (NodThera/Roche), are currently undergoing clinical trials. The ultimate trial data are highly anticipated.

More encouraging findings come from fenamate non-steroidal anti-inflammatory drugs (NSAIDs). These compounds have been approved by the FDA for other diseases’ treatments and also act as selective inhibitors of NLRP3 inflammasome (Daniels et al., 2016). It showed that treatment with mefenamic acid was protective against Aβ-induced memory impairment and NLRP3-mediated neuroinflammation in AD animal models (Daniels et al., 2016). These promising discoveries in animal studies of FDA-approved drugs may enable faster breakthroughs in clinical trials.

Interestingly, many Chinese traditional medicine ingredients and botanical extracts were demonstrated to inhibit NLRP3 inflammasome activation. Resveratrol is a polyphenol complex derived from natural plants, mainly in red grape skins and wine. Several studies have revealed the beneficial effects of resveratrol in the treatment of AD (Qi et al., 2019), cerebral ischemia (He et al., 2017), and SAH (Zhang et al., 2017), all of which are closely related to the modulation of NLRP3 inflammasome. Dl-3-n-butylphthalide (Dl-NBP) is the active ingredient extracted from Chinese herbal celery seeds. It has long been used for the treatment of ischemic stroke. Recently, Dl-NBP is thought to be a novel application for neurodegenerative diseases. In APP/PS1 mice, Dl-NBP treatment reduced TXNIP-NLRP3 interaction and ameliorated neuronal apoptosis by up-regulating Nrf2 to inhibit NLRP3 inflammasome-mediated inflammatory damage, thereby improving oxidative stress injury (Wang et al., 2019). In a Parkinson’s disease model, dl-3-butylphthalide salvaged dopaminergic neurons by inhibiting NLRP3 inflammasome and alleviating mitochondrial damages (Que et al., 2021). Given the low toxicity and fewer side effects of herbal medicine, it may bring a new perspective to the treatment of CNS disease.

### 4.4 Strength and limitations

Compared to traditional literature reviews, analysis with the bibliometric tool CiteSpace yields better insight into the progression of research hotspots and trends, and presents a relatively comprehensive and objective data analysis. Our study is the first bibliometric analysis of NLRP3 inflammasome in neurological diseases, but there are still some limitations. Firstly, the literature data were accessed through the WoSCC database, but with the continuous updating of the database, the results of this study differed somewhat from the actual number of literature currently available. Secondly, the searching topics were only selected to be present in the title, abstract, and keywords, while related terms in the text were not retained and evaluated due to the technical limitations of Web of Science. In addition, only articles and reviews were selected for this study. Meanwhile, the quality of the publications collected was mixed. The reasons mentioned above may make our analysis less comprehensive. Apart from several limitations, we believe that our visualization analysis still provides scholars with a quick understanding of the overall research status and frontiers in the field.

## 5 Conclusion

This study systematically evaluated the role of NLRP3 inflammasome in CNS disorders using bibliometric analysis through 1,217 papers from the WoSCC database during the last decade. Based on the research, China contributed the most in the number of publications, while Western scholars, represented by Prof. Heneka MT, are more influential in this field. In conclusion, the critical role of NLRP3 inflammasome in CNS diseases is well appreciated. Several compounds that interfere with the NLRP3 inflammasome pathway have shown therapeutic benefits in animal models and preclinical trials of neurological disorders. However, further research is needed to improve the efficacy of small molecule NLRP3 inhibitors, reduce their side effects, and make them available for treating CNS illnesses. With the consideration of safety and fewer side effects, traditional Chinese medicine, and plant-derived ingredients many bring new perspectives and alternative options for intractable conditions. Moreover, despite decades of research, our knowledge of NLRP3 inflammasome in CNS diseases is still poor. A better understanding of the underlying mechanisms regulating NLRP3 inflammasome will help us better evaluate the therapeutic potential of targeting NLRP3 inflammasome functions in neurological disorders. We expect that targeting NLRP3 inflammasome may be a promising approach for future therapeutic interventions and would soon be applied to clinical practice to benefit more patients.

## Data Availability

The original contributions presented in the study are included in the article/supplementary material, further inquiries can be directed to the corresponding author.

## References

[B1] Aganna E. Martinon F. Hawkins P. N. Ross J. B. Swan D. C. Booth D. R. (2002). Association of mutations in the NALP3/CIAS1/PYPAF1 gene with a broad phenotype including recurrent fever, cold sensitivity, sensorineural deafness, and AA amyloidosis. Arthritis Rheum. 46 (9), 2445–2452. 10.1002/art.10509 12355493

[B2] Agostini L. Martinon F. Burns K. Mcdermott M. F. Hawkins P. N. Tschopp J. (2004). NALP3 forms an IL-1beta-processing inflammasome with increased activity in Muckle-Wells autoinflammatory disorder. Immunity 20 (3), 319–325. 10.1016/s1074-7613(04)00046-9 15030775

[B3] Aksentijevich I. Nowak M. Mallah M. Chae J. J. Watford W. T. Hofmann S. R. (2002). De novo CIAS1 mutations, cytokine activation, and evidence for genetic heterogeneity in patients with neonatal-onset multisystem inflammatory disease (NOMID): a new member of the expanding family of pyrin-associated autoinflammatory diseases. Arthritis Rheum. 46 (12), 3340–3348. 10.1002/art.10688 12483741 PMC4556432

[B4] Alzheimer's Disease International (2022). 2022 Alzheimer's disease facts and figures. Alzheimer's dement. 18 (4), 700–789. 10.1002/alz.12638 35289055

[B5] Barrington J. Lemarchand E. Allan S. M. (2017). A brain in flame; do inflammasomes and pyroptosis influence stroke pathology? Brain Pathol. 27 (2), 205–212. 10.1111/bpa.12476 27997059 PMC8028888

[B6] Bauernfeind F. G. Horvath G. Stutz A. Alnemri E. S. Macdonald K. Speert D. (2009). Cutting edge: NF-kappaB activating pattern recognition and cytokine receptors license NLRP3 inflammasome activation by regulating NLRP3 expression. J. Immunol. 183 (2), 787–791. 10.4049/jimmunol.0901363 19570822 PMC2824855

[B7] Bellut M. Papp L. Bieber M. Kraft P. Stoll G. Schuhmann M. K. (2021). NLPR3 inflammasome inhibition alleviates hypoxic endothelial cell death *in vitro* and protects blood-brain barrier integrity in murine stroke. Cell Death Dis. 13 (1), 20. 10.1038/s41419-021-04379-z 34930895 PMC8688414

[B8] Broz P. Dixit V. M. (2016). Inflammasomes: Mechanism of assembly, regulation and signalling. Nat. Rev. Immunol. 16 (7), 407–420. 10.1038/nri.2016.58 27291964

[B9] Calabrese L. H. (2002). Anakinra treatment of patients with rheumatoid arthritis. Ann. Pharmacother. 36 (7-8), 1204–1209. 10.1345/aph.1A396 12086555

[B10] Campbell B. C. V. Silva D. A. D. Macleod M. R. Coutts S. B. Schwamm L. H. Davis S. M. (2019). Ischaemic stroke. Nat. Rev. Dis. Prim. 5 (1), 70. 10.1038/s41572-019-0118-8 31601801

[B11] Chen J. Chen Z. J. (2018). PtdIns4P on dispersed trans-Golgi network mediates NLRP3 inflammasome activation. Nature 564 (7734), 71–76. 10.1038/s41586-018-0761-3 30487600 PMC9402428

[B12] Chen C. Song M. (2019). Visualizing a field of research: a methodology of systematic scientometric reviews. PloS one 14 (10), 0223994. 10.1371/journal.pone.0223994 PMC6822756 31671124

[B13] Chen S. Ma Q. Krafft P. R. Hu Q. Rolland W. 2. Sherchan P. (2013). P2X7R/cryopyrin inflammasome axis inhibition reduces neuroinflammation after SAH. Neurobiol. Dis. 58, 296–307. 10.1016/j.nbd.2013.06.011 23816751 PMC3771387

[B14] Coll R. C. Robertson A. A. B. Chae J. J. Higgins S. C. Munoz-planillo R. Inserra M. C. (2015). A small-molecule inhibitor of the NLRP3 inflammasome for the treatment of inflammatory diseases. Nat. Med. 21 (3), 248–255. 10.1038/nm.3806 25686105 PMC4392179

[B15] Daniels M. J. D. Rivers-auty J. Schilling T. Spencer N. G. Watremez W. Fasolino V. (2016). Fenamate NSAIDs inhibit the NLRP3 inflammasome and protect against Alzheimer's disease in rodent models. Nat. Commun. 7, 12504. 10.1038/ncomms12504 27509875 PMC4987536

[B16] Di A. Xiong S. Ye Z. Malireddi R. K. S. Kometani S. Zhong M. (2018). The TWIK2 potassium efflux channel in macrophages mediates NLRP3 inflammasome-induced inflammation. Immunity 49 (1), 56–65.e4. 10.1016/j.immuni.2018.04.032 29958799 PMC6051907

[B17] Dodd W. S. Noda I. Martinez M. Hosaka K. Hoh B. L. (2021). NLRP3 inhibition attenuates early brain injury and delayed cerebral vasospasm after subarachnoid hemorrhage. J. Neuroinflammation 18 (1), 163. 10.1186/s12974-021-02207-x 34284798 PMC8293512

[B18] Dostert C. Petrilli V. Bruggen R. V. Steele C. Mossman B. T. Tschopp J. (2008). Innate immune activation through Nalp3 inflammasome sensing of asbestos and silica. Science 320 (5876), 674–677. 10.1126/science.1156995 18403674 PMC2396588

[B19] Fann D. Y. Lee S. Y. Manzanero S. Tang S. C. Gelderblom M. Chunduri P. (2013a). Intravenous immunoglobulin suppresses NLRP1 and NLRP3 inflammasome-mediated neuronal death in ischemic stroke. Cell Death Dis. 4, 790. 10.1038/cddis.2013.326 PMC3789184 24008734

[B20] Fann D. Y. Lee S. Manzanero S. Chunduri P. Sobey C. G. Arumugam T. V. (2013b). Pathogenesis of acute stroke and the role of inflammasomes. Ageing Res. Rev. 12 (4), 941–966. 10.1016/j.arr.2013.09.004 24103368

[B21] Fann D. Y. Lim Y. Cheng Y. Lok K. Chunduri P. Baik S. (2018). Evidence that NF-κB and MAPK signaling promotes NLRP inflammasome activation in neurons following ischemic stroke. Mol. Neurobiol. 55 (2), 1082–1096. 10.1007/s12035-017-0394-9 28092085

[B22] Fekete C. Vastagh C. Denes A. Hrabovszky E. Nyiri G. Kallo I. (2019). Chronic amyloid beta oligomer infusion evokes sustained inflammation and microglial changes in the rat Hippocampus via NLRP3. Neuroscience 405, 35–46. 10.1016/j.neuroscience.2018.02.046 29522854

[B23] Feng L. Chen Y. Ding R. Fu Z. Yang S. Deng X. (2015). P2X7R blockade prevents NLRP3 inflammasome activation and brain injury in a rat model of intracerebral hemorrhage: involvement of peroxynitrite. J. Neuroinflammation 12, 190. 10.1186/s12974-015-0409-2 26475134 PMC4609067

[B24] Feng Y. Tan Z. Wang M. Xing Y. Dong F. Zhang F. (2020). Inhibition of NLRP3 inflammasome: a prospective target for the treatment of ischemic stroke. Front. Cell. Neurosci. 14, 155. 10.3389/fncel.2020.00155 32581721 PMC7283578

[B25] Flores J. Noel A. Foveau B. Lynham J. Lecrux C. Leblanc A. C. (2018). Caspase-1 inhibition alleviates cognitive impairment and neuropathology in an Alzheimer's disease mouse model. Nat. Commun. 9 (1), 3916. 10.1038/s41467-018-06449-x 30254377 PMC6156230

[B26] Franchi L. Eigenbrod T. Nunez G. (2009). Cutting edge: TNF-alpha mediates sensitization to ATP and silica via the NLRP3 inflammasome in the absence of microbial stimulation. J. Immunol. 183 (2), 792–796. 10.4049/jimmunol.0900173 19542372 PMC2754237

[B27] Galloway J. B. Hyrich K. L. Mercer L. K. Dixon W. G. Watson K. D. Lunt M. (2011). The risk of serious infections in patients receiving anakinra for rheumatoid arthritis: results from the British society for rheumatology biologics register. Rheumatol. Oxf. 50 (7), 1341–1342. 10.1093/rheumatology/ker146 21489973

[B28] Gong Z. Pan J. Shen Q. Li M. Peng Y. (2018). Mitochondrial dysfunction induces NLRP3 inflammasome activation during cerebral ischemia/reperfusion injury. J. Neuroinflammation 15 (1), 242. 10.1186/s12974-018-1282-6 30153825 PMC6114292

[B29] Granowitz E. V. Porat R. Mier J. W. Pribble J. P. Stiles D. M. Bloedow D. C. (1992). Pharmacokinetics, safety and immunomodulatory effects of human recombinant interleukin-1 receptor antagonist in healthy humans. Cytokine 4 (5), 353–360. 10.1016/1043-4666(92)90078-6 1420996

[B30] Guo C. Fulp J. W. Jiang Y. Li X. Chojnacki J. E. Wu J. (2017). Development and characterization of a hydroxyl-sulfonamide analogue, 5-chloro-N-[2-(4-hydroxysulfamoyl-phenyl)-ethyl]-2-methoxy-benzamide, as a novel NLRP3 inflammasome inhibitor for potential treatment of multiple sclerosis. ACS Chem. Neurosci. 8 (10), 2194–2201. 10.1021/acschemneuro.7b00124 28653829 PMC5672903

[B31] Halle A. Hornung V. Petzold G. C. Stewart C. R. Monks B. G. Reinheckel T. (2008). The NALP3 inflammasome is involved in the innate immune response to amyloid-beta. Nat. Immunol. 9 (8), 857–865. 10.1038/ni.1636 18604209 PMC3101478

[B32] He Y. Hara H. Nunez G. (2016). Mechanism and regulation of NLRP3 inflammasome activation. Trends biochem. Sci. 41 (12), 1012–1021. 10.1016/j.tibs.2016.09.002 27669650 PMC5123939

[B33] He Q. Li Z. Wang Y. Hou Y. Li L. Zhao J. (2017). Resveratrol alleviates cerebral ischemia/reperfusion injury in rats by inhibiting NLRP3 inflammasome activation through Sirt1-dependent autophagy induction. Int. Immunopharmacol. 50, 208–215. 10.1016/j.intimp.2017.06.029 28683365

[B34] Heneka M. T. Kummer M. P. Stutz A. Delekate A. Schwartz S. Vieira-saecker A. (2013). NLRP3 is activated in Alzheimer's disease and contributes to pathology in APP/PS1 mice. Nature 493 (7434), 674–678. 10.1038/nature11729 23254930 PMC3812809

[B35] Hoffman H. M. Mueller J. L. Broide D. H. Wanderer A. A. Kolodner R. D. (2001). Mutation of a new gene encoding a putative pyrin-like protein causes familial cold autoinflammatory syndrome and Muckle-Wells syndrome. Nat. Genet. 29 (3), 301–305. 10.1038/ng756 11687797 PMC4322000

[B36] Hornung V. Bauernfeind F. Halle A. Samstad E. O. Kono H. Rock K. L. (2008). Silica crystals and aluminum salts activate the NALP3 inflammasome through phagosomal destabilization. Nat. Immunol. 9 (8), 847–856. 10.1038/ni.1631 18604214 PMC2834784

[B37] Hou Y. Wang Y. He Q. Li L. Xie H. Zhao Y. (2018). Nrf2 inhibits NLRP3 inflammasome activation through regulating Trx1/TXNIP complex in cerebral ischemia reperfusion injury. Behav. Brain Res. 336, 32–39. 10.1016/j.bbr.2017.06.027 28851669

[B38] Imani D. Azimi A. Salehi Z. Rezaei N. Emamnejad R. Sadr M. (2018). Association of nod-like receptor protein-3 single nucleotide gene polymorphisms and expression with the susceptibility to relapsing-remitting multiple sclerosis. Int. J. Immunogenet. 45 (6), 329–336. 10.1111/iji.12401 30264444

[B39] Inoue M. Williams K. L. Gunn M. D. Shinohara M. L. (2012a). NLRP3 inflammasome induces chemotactic immune cell migration to the CNS in experimental autoimmune encephalomyelitis. Proc. Natl. Acad. Sci. U. S. A. 109 (26), 10480–10485. 10.1073/pnas.1201836109 22699511 PMC3387125

[B40] Inoue M. Williams K. L. Oliver T. Vandenabeele P. Rajan J. V. Miao E. A. (2012b). Interferon-beta therapy against EAE is effective only when development of the disease depends on the NLRP3 inflammasome. Sci. Signal. 5 (225), ra38. 10.1126/scisignal.2002767 22623753 PMC3509177

[B41] Irrera N. Pizzino G. Calo M. Pallio G. Mannino F. Fama F. (2017). Lack of the Nlrp3 inflammasome improves mice recovery following traumatic brain injury. Front. Pharmacol. 8, 459. 10.3389/fphar.2017.00459 28769794 PMC5509758

[B42] Ising C. Venegas C. Zhang S. Scheiblich H. Schmidt S. V. Vieira-saecker A. (2019). NLRP3 inflammasome activation drives tau pathology. Nature 575 (7784), 669–673. 10.1038/s41586-019-1769-z 31748742 PMC7324015

[B43] Ismael S. Nasoohi S. Ishrat T. (2018). MCC950, the selective inhibitor of nucleotide oligomerization domain-like receptor protein-3 inflammasome, protects mice against traumatic brain injury. J. Neurotrauma 35 (11), 1294–1303. 10.1089/neu.2017.5344 29295651 PMC5962912

[B44] Iyer S. S. He Q. Janczy J. R. Elliott E. I. Zhong Z. Olivier A. K. (2013). Mitochondrial cardiolipin is required for Nlrp3 inflammasome activation. Immunity 39 (2), 311–323. 10.1016/j.immuni.2013.08.001 23954133 PMC3779285

[B45] Jesus A. A. Goldbach-mansky R. (2014). IL-1 blockade in autoinflammatory syndromes. Annu. Rev. Med. 65, 223–244. 10.1146/annurev-med-061512-150641 24422572 PMC4178953

[B46] Jha S. Srivastava S. Y. Brickey W. J. Iocca H. Toews A. Morrison J. P. (2010). The inflammasome sensor, NLRP3, regulates CNS inflammation and demyelination via caspase-1 and interleukin-18. J. Neurosci. 30 (47), 15811–15820. 10.1523/JNEUROSCI.4088-10.2010 21106820 PMC6633756

[B47] Juliana C. Fernandes-alnemri T. Wu J. Datta P. Solorzano L. Yu J. (2010). Anti-inflammatory compounds parthenolide and Bay 11-7082 are direct inhibitors of the inflammasome. J. Biol. Chem. 285 (13), 9792–9802. 10.1074/jbc.M109.082305 20093358 PMC2843228

[B48] Kayagaki N. Warming S. Lamkanfi M. Walle L. V. Louie S. Dong J. (2011). Non-canonical inflammasome activation targets caspase-11. Nature 479 (7371), 117–121. 10.1038/nature10558 22002608

[B49] Kayagaki N. Stowe I. B. Lee B. L. O'rourke K. Anderson K. Warming S. (2015). Caspase-11 cleaves gasdermin D for non-canonical inflammasome signalling. Nature 526 (7575), 666–671. 10.1038/nature15541 26375259

[B50] Lalor S. J. Dungan L. S. Sutton C. E. Basdeo S. A. Fletcher J. M. Mills K. H. G. (2011). Caspase-1-processed cytokines IL-1beta and IL-18 promote IL-17 production by gammadelta and CD4 T cells that mediate autoimmunity. J. Immunol. 186 (10), 5738–5748. 10.4049/jimmunol.1003597 21471445

[B51] Li C. Lin H. He H. Ma M. Jiang W. Zhou R. (2022). Inhibition of the NLRP3 inflammasome activation by manoalide ameliorates experimental autoimmune encephalomyelitis pathogenesis. Front. Cell Dev. Biol. 10, 822236. 10.3389/fcell.2022.822236 35252186 PMC8888861

[B52] Liston A. Masters S. L. (2017). Homeostasis-altering molecular processes as mechanisms of inflammasome activation. Nat. Rev. Immunol. 17 (3), 208–214. 10.1038/nri.2016.151 28163301

[B53] Liu X. Zhang X. Ding Y. Zhou W. Tao L. Lu P. (2017). Nuclear factor E2-related factor-2 negatively regulates NLRP3 inflammasome activity by inhibiting reactive oxygen species-induced NLRP3 priming. Antioxid. Redox Signal. 26 (1), 28–43. 10.1089/ars.2015.6615 27308893 PMC5198158

[B54] Liu Q. Zhang D. Hu D. Zhou X. Zhou Y. (2018). The role of mitochondria in NLRP3 inflammasome activation. Mol. Immunol. 103, 115–124. 10.1016/j.molimm.2018.09.010 30248487

[B55] Luciunaite A. Mcmanus R. M. Jankunec M. Racz I. Dansokho C. Dalgediene I. (2020). Soluble Aβ oligomers and protofibrils induce NLRP3 inflammasome activation in microglia. J. Neurochem. 155 (6), 650–661. 10.1111/jnc.14945 31872431

[B56] Luo Y. Lu J. Ruan W. Guo X. Chen S. (2019a). MCC950 attenuated early brain injury by suppressing NLRP3 inflammasome after experimental SAH in rats. Brain Res. Bull. 146, 320–326. 10.1016/j.brainresbull.2019.01.027 30716395

[B57] Luo Y. Reis C. Chen S. (2019b). NLRP3 inflammasome in the pathophysiology of hemorrhagic stroke: A review. Curr. Neuropharmacol. 17 (7), 582–589. 10.2174/1570159X17666181227170053 30592254 PMC6712291

[B58] Mackenzie S. H. Schipper J. L. Clark A. C. (2010). The potential for caspases in drug discovery. Curr. Opin. Drug Discov. Devel. 13 (5), 568–576. PMC3289102 20812148

[B59] Malhotra S. Rio J. Urcelay E. Nurtdinov R. Bustamante M. F. Fernandez O. (2015). NLRP3 inflammasome is associated with the response to IFN-beta in patients with multiple sclerosis. Brain 138, 644–652. 10.1093/brain/awu388 25586466 PMC4408432

[B60] Malhotra S. Sorosina M. Rio J. Peroni S. Midaglia L. Villar L. M. (2018). NLRP3 polymorphisms and response to interferon-beta in multiple sclerosis patients. Mult. Scler. 24 (11), 1507–1510. 10.1177/1352458517739137 29117789

[B61] Malhotra S. Costa C. Eixarch H. Keller C. W. Amman L. Martinez-banaclocha H. (2020). NLRP3 inflammasome as prognostic factor and therapeutic target in primary progressive multiple sclerosis patients. Brain 143 (5), 1414–1430. 10.1093/brain/awaa084 32282893

[B62] Martinon F. Burns K. Tschopp J. (2002). The inflammasome: a molecular platform triggering activation of inflammatory caspases and processing of proIL-beta. Mol. Cell 10 (2), 417–426. 10.1016/s1097-2765(02)00599-3 12191486

[B63] Mckenzie B. A. Mamik M. K. Saito L. B. Boghozian R. Monaco M. C. Major E. O. (2018). Caspase-1 inhibition prevents glial inflammasome activation and pyroptosis in models of multiple sclerosis. Proc. Natl. Acad. Sci. U. S. A. 115 (26), E6065–E6074. 10.1073/pnas.1722041115 29895691 PMC6042136

[B64] Mills E. L. Ryan D. G. Prag H. A. Dikovskaya D. Menon D. Zaslona Z. (2018). Itaconate is an anti-inflammatory metabolite that activates Nrf2 via alkylation of KEAP1. Nature 556 (7699), 113–117. 10.1038/nature25986 29590092 PMC6047741

[B65] Minutoli L. Puzzolo D. Rinaldi M. Irrera N. Marini H. Arcoraci V. (2016). ROS-mediated NLRP3 inflammasome activation in brain, heart, kidney, and testis ischemia/reperfusion injury. Oxid. Med. Cell. Longev. 2016, 2183026. 10.1155/2016/2183026 27127546 PMC4835650

[B66] Munoz-planillo R. Kuffa P. Martinez-colon G. Smith B. L. Rajendiran T. M. Nunez G. (2013). K⁺ efflux is the common trigger of NLRP3 inflammasome activation by bacterial toxins and particulate matter. Immunity 38 (6), 1142–1153. 10.1016/j.immuni.2013.05.016 23809161 PMC3730833

[B67] Murakami T. Ockinger J. Yu J. Byles V. Mccoll A. Hofer A. M. (2012). Critical role for calcium mobilization in activation of the NLRP3 inflammasome. Proc. Natl. Acad. Sci. U. S. A. 109 (28), 11282–11287. 10.1073/pnas.1117765109 22733741 PMC3396518

[B68] O'brien W. T. Pham L. Symons G. F. Monif M. Shultz S. R. Mcdonald S. J. (2020). The NLRP3 inflammasome in traumatic brain injury: potential as a biomarker and therapeutic target. J. Neuroinflammation 17 (1), 104. 10.1186/s12974-020-01778-5 32252777 PMC7137518

[B69] Panicker N. Sarkar S. Harischandra D. S. Neal M. Kam T. Jin H. (2019). Fyn kinase regulates misfolded alpha-synuclein uptake and NLRP3 inflammasome activation in microglia. J. Exp. Med. 216 (6), 1411–1430. 10.1084/jem.20182191 31036561 PMC6547864

[B70] Pendlebury S. T. Rothwell P. M. (2009). Prevalence, incidence, and factors associated with pre-stroke and post-stroke dementia: a systematic review and meta-analysis. Lancet. Neurol. 8 (11), 1006–1018. 10.1016/S1474-4422(09)70236-4 19782001

[B71] Qi Y. Klyubin I. Cuello A. C. Rowan M. J. (2018). NLRP3-dependent synaptic plasticity deficit in an Alzheimer's disease amyloidosis model *in vivo* . Neurobiol. Dis. 114, 24–30. 10.1016/j.nbd.2018.02.016 29477641

[B72] Qi Y. Shang L. Liao Z. Su H. Jing H. Wu B. (2019). Intracerebroventricular injection of resveratrol ameliorated Aβ-induced learning and cognitive decline in mice. Metab. Brain Dis. 34 (1), 257–266. 10.1007/s11011-018-0348-6 30460524

[B73] Que R. Zheng J. Chang Z. Zhang W. Li H. Xie Z. (2021). Dl-3-n-Butylphthalide rescues dopaminergic neurons in Parkinson's disease models by inhibiting the NLRP3 inflammasome and ameliorating mitochondrial impairment. Front. Immunol. 12, 794770. 10.3389/fimmu.2021.794770 34925379 PMC8671881

[B74] Ramirez J. Canete J. D. (2018). Anakinra for the treatment of rheumatoid arthritis: a safety evaluation. Expert Opin. Drug Saf. 17 (7), 727–732. 10.1080/14740338.2018.1486819 29883212

[B75] Ren H. Kong Y. Liu Z. Zang D. Yang X. Wood K. (2018). Selective NLRP3 (pyrin domain-containing protein 3) inflammasome inhibitor reduces brain injury after intracerebral hemorrhage. Stroke 49 (1), 184–192. 10.1161/STROKEAHA.117.018904 29212744 PMC5753818

[B76] Rossi-semerano L. Fautrel B. Wendling D. Hachulla E. Galeotti C. Semerano L. (2015). Tolerance and efficacy of off-label anti-interleukin-1 treatments in France: a nationwide survey. Orphanet J. Rare Dis. 10, 19. 10.1186/s13023-015-0228-7 25758134 PMC4340831

[B77] Ruan Y. Qiu X. Lv Y. Dong D. Wu X. Zhu J. (2019). Kainic acid Induces production and aggregation of amyloid β-protein and memory deficits by activating inflammasomes in NLRP3- and NF-κB-stimulated pathways. Aging 11 (11), 3795–3810. 10.18632/aging.102017 31182681 PMC6594814

[B78] Shi J. Zhao Y. Wang Y. Gao W. Ding J. Li P. (2014). Inflammatory caspases are innate immune receptors for intracellular LPS. Nature 514 (7521), 187–192. 10.1038/nature13683 25119034

[B79] Shimada K. Crother T. R. Karlin J. Dagvadorj J. Chiba N. Chen S. (2012). Oxidized mitochondrial DNA activates the NLRP3 inflammasome during apoptosis. Immunity 36 (3), 401–414. 10.1016/j.immuni.2012.01.009 22342844 PMC3312986

[B80] Stancu I. Cremers N. Vanrusselt H. Couturier J. Vanoosthuyse A. Kessels S. (2019). Aggregated Tau activates NLRP3-ASC inflammasome exacerbating exogenously seeded and non-exogenously seeded Tau pathology *in vivo* . Acta Neuropathol. 137 (4), 599–617. 10.1007/s00401-018-01957-y 30721409 PMC6426830

[B81] Thompson A. J. Baranzini S. E. Geurts J. Hemmer B. Ciccarelli O. (2018). Multiple sclerosis. Lancet 391 (10130), 1622–1636. 10.1016/S0140-6736(18)30481-1 29576504

[B82] Venegas C. Kumar S. Franklin B. S. Dierkes T. Brinkschulte R. Tejera D. (2017). Microglia-derived ASC specks cross-seed amyloid-beta in Alzheimer's disease. Nature 552 (7685), 355–361. 10.1038/nature25158 29293211

[B83] Walsh J. G. Muruve D. A. Power C. (2014). Inflammasomes in the CNS. Nat. Rev. Neurosci. 15 (2), 84–97. 10.1038/nrn3638 24399084

[B84] Wang Q. Tang X. N. Yenari M. A. (2007). The inflammatory response in stroke. J. Neuroimmunol. 184 (1-2), 53–68. 10.1016/j.jneuroim.2006.11.014 17188755 PMC1868538

[B85] Wang C. Xu Y. Wang X. Guo C. Wang T. Wang Z. (2019). Dl-3-n-Butylphthalide inhibits NLRP3 inflammasome and mitigates alzheimer's-like pathology via nrf2-TXNIP-TrX Axis. Antioxid. Redox Signal. 30 (11), 1411–1431. 10.1089/ars.2017.7440 29634349

[B86] Ward R. Li W. Abdul Y. Jackson L. Dong G. Jamil S. (2019). NLRP3 inflammasome inhibition with MCC950 improves diabetes-mediated cognitive impairment and vasoneuronal remodeling after ischemia. Pharmacol. Res. 142, 237–250. 10.1016/j.phrs.2019.01.035 30818045 PMC6486792

[B87] Xing Y. Yao X. Li H. Xue G. Guo Q. Yang G. (2017). Cutting edge: TRAF6 mediates TLR/IL-1R signaling-induced nontranscriptional priming of the NLRP3 inflammasome. J. Immunol. 199 (5), 1561–1566. 10.4049/jimmunol.1700175 28739881

[B88] Xiong X. Liu L. Yang Q. (2016). Functions and mechanisms of microglia/macrophages in neuroinflammation and neurogenesis after stroke. Prog. Neurobiol. 142, 23–44. 10.1016/j.pneurobio.2016.05.001 27166859

[B89] Xu X. Yin D. Ren H. Gao W. Li F. Sun D. (2018). Selective NLRP3 inflammasome inhibitor reduces neuroinflammation and improves long-term neurological outcomes in a murine model of traumatic brain injury. Neurobiol. Dis. 117, 15–27. 10.1016/j.nbd.2018.05.016 29859317

[B90] Xu P. Hong Y. Xie Y. Yuan K. Li J. Sun R. (2021). TREM-1 exacerbates neuroinflammatory injury via NLRP3 inflammasome-mediated pyroptosis in experimental subarachnoid hemorrhage. Transl. Stroke Res. 12 (4), 643–659. 10.1007/s12975-020-00840-x 32862402

[B91] Yang F. Wang Z. Wei X. Han H. Meng X. Zhang Y. (2014). NLRP3 deficiency ameliorates neurovascular damage in experimental ischemic stroke. J. Cereb. Blood Flow. Metab. 34 (4), 660–667. 10.1038/jcbfm.2013.242 24424382 PMC3982086

[B92] Ye X. Shen T. Hu J. Zhang L. Zhang Y. Bao L. (2017). Purinergic 2X7 receptor/NLRP3 pathway triggers neuronal apoptosis after ischemic stroke in the mouse. Exp. Neurol. 292, 46–55. 10.1016/j.expneurol.2017.03.002 28274860

[B93] Yuan R. Fan H. Cheng S. Gao W. Xu X. Lv S. (2017). Silymarin prevents NLRP3 inflammasome activation and protects against intracerebral hemorrhage. Biomed. Pharmacother. 93, 308–315. 10.1016/j.biopha.2017.06.018 28651232

[B94] Zhang X. Wu Q. Zhang Q. Lu Y. Liu J. Li W. (2017). Resveratrol attenuates early brain injury after experimental subarachnoid hemorrhage via inhibition of NLRP3 inflammasome activation. Front. Neurosci. 11, 611. 10.3389/fnins.2017.00611 29163015 PMC5675880

[B95] Zhong Z. Liang S. Sanchez-lopez E. He F. Shalapour S. Lin X. (2018). New mitochondrial DNA synthesis enables NLRP3 inflammasome activation. Nature 560 (7717), 198–203. 10.1038/s41586-018-0372-z 30046112 PMC6329306

[B96] Zhou R. Yazdi A. S. Menu P. Tschopp J. (2011). A role for mitochondria in NLRP3 inflammasome activation. Nature 469 (7329), 221–225. 10.1038/nature09663 21124315

[B97] Zhou Y. Tong Z. Jiang S. Zheng W. Zhao J. Zhou X. (2020). The roles of endoplasmic reticulum in NLRP3 inflammasome activation. Cells 9 (5), E1219. 10.3390/cells9051219 32423023 PMC7291288

